# Robotic exploration of amino-acid functionalised molybdenum blue polyoxometalate nanoclusters†

**DOI:** 10.1039/d5cc00703h

**Published:** 2025-04-04

**Authors:** Haiyang Guo, Donglin He, De-Liang Long, Leroy Cronin

**Affiliations:** a School of Chemistry, The University of Glasgow University Avenue Glasgow G12 8QQ UK Deliang.Long@glasgow.ac.uk Lee.Cronin@glasgow.ac.uk; b College of Biological, Chemical Sciences and Engineering, Jiaxing University Jiaxing 314001 P. R. China

## Abstract

A robotic platform was employed to investigate the synthetic conditions for the assembly of molybdenum blue (MB) polyoxometalates in a large chemical space that included concentrations of reactants, the amount of reducing agent, solution pH *etc.* Four different solid phases of {Mo_154_} MB clusters were identified and crystallographically explored, demonstrating the robotic platform's strength in targeted exploration.

## Abstract 2

A robotic platform was employed to investigate the synthetic conditions for the assembly of molybdenum blue (MB) polyoxometalates in a large chemical space that included concentrations of reactants, the amount of reducing agent, solution pH *etc.*

## Graphical abstract

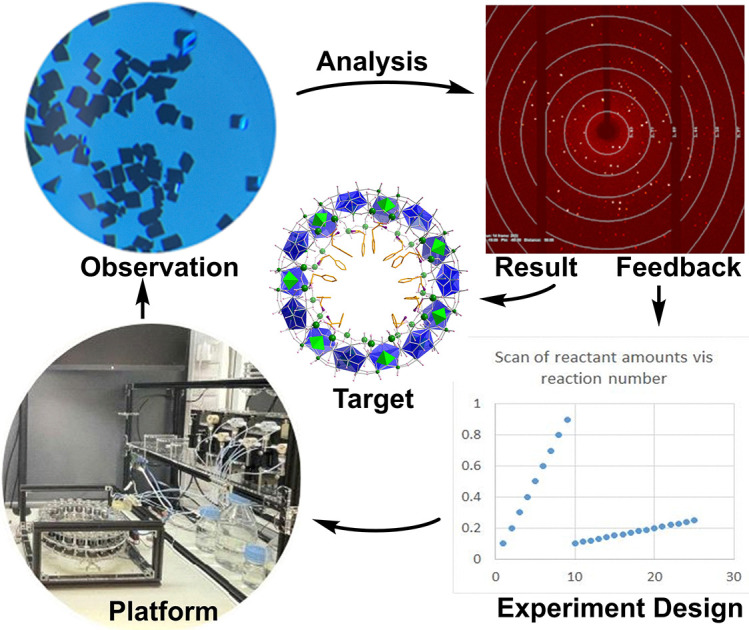

Molecular metal oxides, polyoxometalates (POMs), with diverse structures and properties^1^ play an important role in catalysis,^2^ electronic devices and materials,^3^ POM metal–organic hybrids,^4^ and directed evolution on the subnanometer scale.^5^ Within the POM family, giant “nanosized” polyoxomolybdates with over a hundred molybdenum atoms in a single molecule and size dimensions close to proteins form a unique subgroup, a bridge over the gap between traditional molecular entities and polymeric objects less precisely defined. Giant polyoxomolybdate clusters have unique properties and can be further integrated into larger assemblies and materials with high dimension structures.^6^ Due to their structural complexity and functional diversity, giant molybdenum blue (MB) whorled nanoclusters are of particular interest. In 1996, Müller *et al.* determined the crystal structure of the MB wheel {Mo_154_} for the first time.^7^ Since then, the literature has seen an explosion of research into giant polyoxomolybdates.^8^ Specifically, the work involved the development of MBs with molecular growth,^8*f*,*l*^ lanthanide substitution,^8*i*^ and organic ligand functionalisation.^9^ While amino acid- and peptide-functionalised MBs have been documented in the literature,^8*l*–10^ the systematic exploration of expanded dimensional structures within the MB domain remains limited. In order to produce novel high-dimensional materials with improved properties and functions, the precise modulation of the compositions and molecular structures with amino acids to explore the potential derivatisations requires a search of the chemical space. This is because it is a major challenge to explore the complex multicomponent chemical reactions that lead to new-dimensional products and their discovery requires molecular self-assembly and crystallisation. The discovery of new MBs that are made up of several building blocks and supporting units *via* self-assembly processes or modified by organic ligands needs the multi-parameter control of complicated procedures and requires a large set of reactions. Additionally, many new chemical procedures are not always reproducible, and many of the new compounds have low yields. Recently, we have developed a robotic system known as the modular wheel platform (MWP) for combinatorial exploration within the inorganic chemical space.^11^ This system has proven highly effective in automating the screening of reaction conditions, enabling the discovery of novel and extremely large POMs.^11^ In this work, we constructed a robotic system with the automatic MWP to efficiently explore the formation chemical space of gigantic and novel MB POMs. The robotic platform consists of an MWP (Fig. S1a, ESI†) capable of performing parallel synthesis with up to 24 reactors in an array,^11^ a heating mantle unit (Fig. S1b, ESI†) capable of holding up to 48 reactions and a high-throughput filtration module (Fig. S1c, ESI†) capable of performing 48 filtration operations simultaneously. The MWP has the rotation of the Geneva wheel, which is synchronised with both parallel/sequential liquid dispensing and stirring of reagents to conduct the synthesis efficiently. In addition, the high-precision syringe pumps in the MWP perform liquid handling, mixing, cleaning, dynamic pH detection and sample extraction/transfer. Furthermore, the stock solutions used in the MWP are stored in a temperature-controlled box for the fine-tuning of the reaction conditions to ensure reproducibility.

In this work, we focused on MB clusters modified by amino acids in one-pot reaction; reaction parameters including concentrations of reactants, the pH of the solution and reaction temperature were considered as the main factors for the synthesis. The concentrations of the reactants were adjusted by varying the added volume of stock solution. The pH of the solution was controlled by adding different volumes of HCl stock solution. The reactions were heated to 65 °C by the heating mantle unit (Tables S1–S4, Spaces 1–4, ESI†). The reactions in Space 5 were carried out to test the reaction and crystallisation at room temperature (RT). The reactions in all Spaces were filtered by a high-throughput filtration module before the crystallisation at RT to improve the yield and purity of the product. Subsequently we checked the purity and confirmed the structure of the product by multiple unit cell checks along with structure determination by single crystal X-ray diffraction (Tables S6–S10, ESI†).

The high-throughput reactions were carried out by the robotic platform in 5 cycles (Spaces 1–5 in Tables S1–S5, ESI†), completing a total of 960 reactions. Fig. 1 summarises the aims, reaction contents and results of five reaction spaces. In each reaction space from second to fifth, the variation in conditions was decided by the operator's feedback based on the results of the former reaction space. In addition, the first 24 reactions from Space 1 (Table S1, ESI†) were repeated once before carrying out new reactions of each cycle in Spaces 2–5 to confirm the repeatability of the synthesis on the robotic platform. In this way, efficient optimization and repeatability of MB cluster synthesis could be achieved with the help of the robotic platform.

**Fig. 1:**
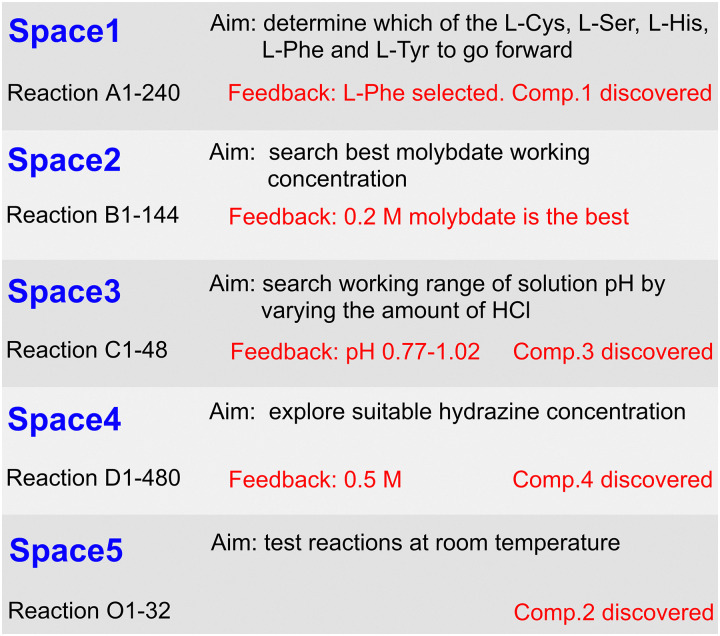
Summary of spaces in terms of aim, feedback and products obtained.

Based on the knowledge of previously reported MB clusters of amino acids,^8*l*–10^ we screened several amino acids (l-cysteine, l-serine, l-histidine, l-phenylalanine and l-tyrosine) as structure-directing ligands in the first cycle of experiments (Table S1, Space 1, ESI†). Single crystals of MB-type {Mo_154_} wheels (compound 1) were successfully obtained using l-phenylalanine (l-Phe) (Fig. 1). As shown in Fig. 2, the resultant {Mo_154_} wheel-like structures demonstrate distinct aromatic ring amino acid-lined cavities, which indicate the significance of the l-Phe for directing the self-assembly of inorganic nanoclusters. Therefore, l-Phe was selected for further studies (Spaces 2–5) to explore expanded chemical spaces for MB formation. The following Spaces 2–5 were designed to develop {Mo_154_} wheels of different compositions as refined reaction conditions by varying component concentrations and the reaction temperature. Space 2 (Fig. 1 and Table S2, ESI†) focused on determining the optimal molybdate working concentration, with 0.2 M identified as the most effective condition for product crystal growth. Space 3 in Table S3 (ESI†) was investigated by varying the working pH range by changing the amount of HCl, revealing an optimal range of pH 0.77–1.02 and resulting in the discovery of compound 3. In addition, 3 under reaction condition C25 in Space 3 was repeated three times to see if it would give high crystal quality (Fig. S3, ESI†) and to further confirm the reliability of the robotic platform. Space 4 shown in Table S4 (ESI†) was explored varying the hydrazine concentration, concluding that 0.5 M was the most suitable, facilitating the identification of compound 4. Space 5 further tested reactions at room temperature, culminating in the discovery of compound 2. Four different solid structures of {Mo_154_} containing l-phenylalanine ligands with different composition and packing models were obtained after all reactions.

**Fig. 2:**
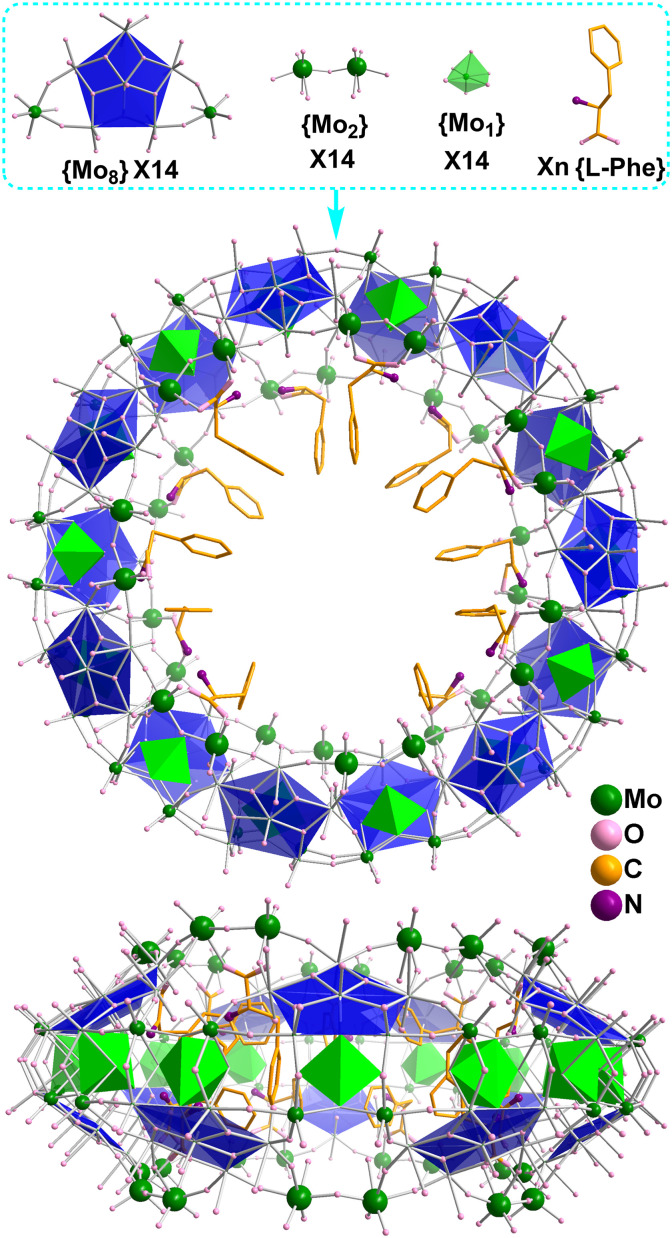
{Mo_154_} frameworks consisting of 14 pentagon based {Mo_8_} building blocks, 14 corner sharing {Mo_2_} dimers and 14 {Mo_1_} backbone supporting units with variable numbers of l-Phe ligands in compounds 1–4.

All four structures (Fig. 3) were found to be MB type {Mo_154_} wheels with phenylalanine as ligands coordinating to {Mo_2_} dimers on the edges of the {Mo_154_} clusters. Each structure has nearly the same {Mo_154_} framework previously reported,^7^ consisting of 14 pentagon based {Mo_8_} building blocks, 14 corner sharing {Mo_2_} dimers and 14 {Mo_1_} backbone supporting units but with slightly different compositions in numbers of phenylalanine ligands and counter sodium cations per cluster verified by chemical analysis. 1 crystallises in the monoclinic system with *C*2 space group and formula Na_6_H_8_[Mo_154_H_14_O_462_(H_2_O)_50_(C_9_H_11_NO_2_)_10_]·180H_2_O. 1 displays isolated clusters which have no Mo–O–Mo covalent bonding bridges between them. The packing diagram in Fig. 3 shows the layer arrangement of the {Mo_154_} wheels in the solid state and the layers are displaced along the crystallographic *a* axis in repeating ABAB patterns. 2 crystallises in the orthorhombic system with *P*2_1_2_1_2 space group and formula Na_4_H_10_[Mo_154_H_14_O_462_(H_2_O)_52_(C_9_H_11_NO_2_)_9_]·230H_2_O. Two types of isolated {Mo_154_} clusters were identified in the asymmetric unit. No connection of Mo–O–Mo bridging bonds between clusters was observed. Molecular packing shows herringbone patterns (Fig. 3). 3 crystallises in the triclinic system with *P*1̄ space group. Two half {Mo_154_} wheel moieties were found in the asymmetric unit. One half moiety extends to complete the isolated {Mo_154_} wheel of formula [Mo_154_O_462_H_14_(H_2_O)_56_(C_9_H_11_NO_2_)_7_]^14−^. The other half moiety expands to complete an unusual {Mo_154_} wheel cluster with formula [Mo_154_O_462_H_14_(H_2_O)_51_(C_9_H_11_NO_2_)_7_]^14−^, which forms a one-dimensional chain with fivefold Mo–O–Mo bridges^8*k*^ between the {Mo_154_} clusters (Fig. 3). The empirical formula of 3 is determined as Na_12_H_16_[Mo_154_O_462_H_14_(H_2_O)_56_(C_9_H_11_NO_2_)_7_][Mo_154_O_462_H_14_(H_2_O)_51_(C_9_H_11_NO_2_)_7_]·460H_2_O. 4 crystallises in the orthorhombic system with *Aea*2 space group and composition Na_11_H_3_[Mo_154_H_14_O_462_(H_2_O)_56_(C_9_H_11_NO_2_)_6_]·200H_2_O. A half {Mo_154_} cluster was found in the asymmetrical unit. Each completed {Mo_154_} cluster links to four neighbouring {Mo_154_} clusters each *via* a Mo–O–Mo single bridge, thus forming a two dimensional layer structure of 4-connected topology.

**Fig. 3:**
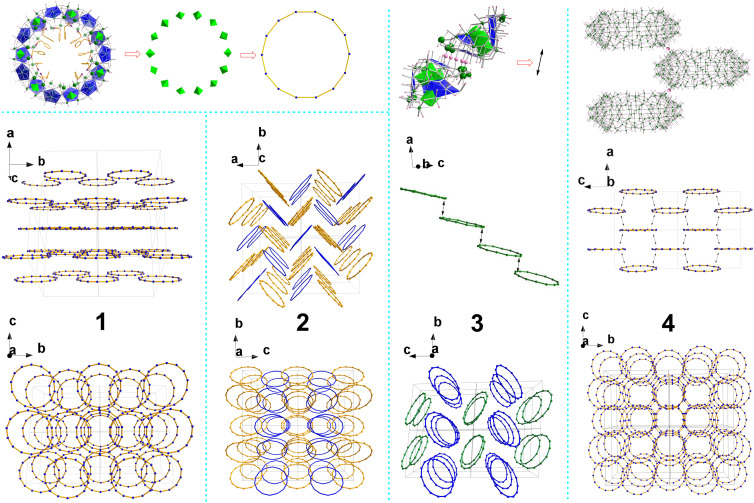
Representative packing diagrams of {Mo_154_} wheels in solid states. {Mo_154_} wheels are symbolised by the ring of the 14 backbone {Mo_1_} centres. 1: (above) layer arrangements; (below) view down layers of displaced ABAB patterns along the crystallographic *a* axis. 2: (above) herringbone pattern arrangements. Yellow and blue colours indicate {Mo_154_} wheels from two different types of isolated {Mo_154_} clusters found in the asymmetric unit; (below) view down along the crystallographic *a* axis. 3: (top) the fivefold Mo–O–Mo bridges between {Mo_154_} wheels illustrated by enlarged O spheres; (middle) 1-D chain with dual arrows indicating the places of the fivefold Mo–O–Mo bridges between {Mo_154_} wheels; (bottom) isolated (blue) and 1-D chain (green) of {Mo_154_} wheels. 4: (top) single bridges between {Mo_154_} wheels illustrated by enlarged O spheres; (middle) 2D layer of wheels on the crystallographic *ac* plane, the dual arrows indicate the places of single Mo–O–Mo bridges between {Mo_154_} wheels; (bottom) view along the crystallographic *a* axis.

All four structures consist of {Mo_154_} type clusters with slightly different compositions in numbers of l-Phe ligands and counter sodium cations verified by chemical analysis. We propose that the different compositions and extending structures primarily arise from the solution pH during the reaction and crystallisation processes, which influences the degree of protonation on the clusters and the ease of carboxylate coordination to the Mo centres. The degree of protonation determines the number of sodium cations needed for charge balance, and the varying number of sodium cations bridge the clusters differently, directing the distinct molecular packing arrangement. Additionally, a high proton concentration facilitates the condensation process, promoting the formation of Mo–O–Mo bridges that link clusters within the crystals. As the main parts in all four structures are {Mo_154_}, it is meaningful to compare the volumes per {Mo_154_} cluster (VPC) in the crystals, which are listed in Table S10 (ESI†). Smaller VPC indicates denser molecular packing and closer inter-cluster interaction or more covalent Mo–O–Mo bonding between clusters. 1 and 2 are isolated clusters with only hydrogen bonding or sodium cation connection between clusters. 1 has smaller VPC than 2 and it is seen that 1 has more efficient packing in parallel ring layer mode while 2 has a less efficient herringbone arrangement. 2 has the biggest VPC with the least number of Na^+^ cations among the four structures. 3 also has a herringbone arrangement with half {Mo_154_} clusters as isolated and the other half forming 1-D chain structures. 4 forms a covalently connected 2-D layer which has the smallest VPC among the four structures.

Using this automated platform, we identified 4 novel phases formed by MB type {Mo_154_} clusters, and achieved reproducible synthesis of {Mo_154_} with varying numbers of l-Phe ligands. These structures, which are challenging to obtain through random synthesis, demonstrate the robotic platform's strength in targeted exploration. Each compound was isolated *via* crystallization and, importantly, can be reliably reproduced using our modular wheeled platform. Furthermore, the macrocycle structure of {Mo_154_} MB enables the formation of diverse architectures, including 0D units, 1D arrays, and 2D mesh extensions, highlighting its structural versatility.

This work was supported by the EPSRC (no. EP/L023652/1; EP/R009902/1; EP/R020914/1; EP/R01308X/1; EP/S017046/1; EP/S019472/1; EP/V048341/1), the European Research Council (Project 670467 SMART-POM) and the University of Glasgow. We thank the Diamond Light Source for machine time on Beamline I19, under the proposal CY30458.

## Data availability

The data used in this manuscript is included in the ESI.†

## Conflicts of interest

There are no conflicts of interest to declare.

## Supplementary Material

CC-061-D5CC00703H-s001

CC-061-D5CC00703H-s002
