# Supplementary material for: Robotic exploration of amino-acid functionalised molybdenum blue polyoxometalate nanoclusters
Source: Chem Commun (Camb). 2025 Apr 4;61(39):7121–4. doi: 10.1039/d5cc00703h (PMC12004214; doi:10.1039/d5cc00703h)
Supplement: CC-061-D5CC00703H-s001 [file CC-061-D5CC00703H-s001.pdf]

This journal is © The Royal Society of Chemistry 2025

Supplementary Information

Robotic exploration of amino-acid functionalised Molybdenum Blue polyoxometalate nanoclusters

Haiyang Guo,[a,b] Donglin He,[a] De-Liang Long,*[a] and Leroy Cronin*[a]

[a] School of Chemistry, The University of Glasgow, University Avenue, Glasgow, G12 8QQ, UK.
E-mail: Deliang.Long@glasgow.ac.uk, Lee.Cronin@glasgow.ac.uk

[b] College of Biological, Chemical Sciences and Engineering, Jiaxing University, Jiaxing, 314001, P. R. China

Table of Contents

1. Materials.......... 3

2. Instrumentation and Methods 3

2.1 Elemental analysis 3

2.2 Inductively coupled plasma optical emission spectrometry (ICP-OES).......... 3

2.3 Thermogravimetric Analysis (TGA) 3

2.4 FT- IR Spectroscopy.......... 3

2.5 Single Crystal X-Ray Diffraction.......... 3

2.6 pH Measurements.......... 4

2.7 Robotic platform.......... 4

3. Programmed Searching Procedures.......... 6

3.1 Reaction conditions and chemical Spaces 1, 2, 3 and 4.......... 6

3.2 Reaction conditions and chemical Space 5 39

4. Optimised Synthetic Procedures.......... 41

4.1 Compound 1 {Mo_{154}(L-Phe)$_{10}$} – Initial Discovery 41

4.2 Compound 1 {Mo_{154}(L-Phe)$_{10}$} – Optimal Reactant 41

4.3 Compound 2 {Mo_{154}(L-Phe)$_{9}$} 42

4.4 Compound 3 1D {Mo_{154}(L-Phe)$_{7}$} 42

4.5 Compound 4 2D {Mo_{154}(L-Phe)$_{6}$} 43

5. Crystallographic Data.......... 44

6. Structure Analysis and Description.......... 48

7. Characterisations and Supplementary Data 51

7.1 Chemical analysis 51

7.2 Uv-vis Spectra 52

7.3 Thermogravimetric analysis 52

8. References.......... 54

1. Materials

Sodium molybdate dihydrate (≥ 99%) was sourced from Sigma Aldrich and used without further purification. Hydrazine dihydrochloride (≥ 98%) was sourced from Alfa Aesar and used without further purification. Hydrochloric acid (37%), L-cysteine, (97%), L-serine (≥99%), L-histidine (≥99%), L-phenylalanine (≥98%), L-tyrosine (≥98%) were sourced from Sigma-Aldrich. Water used in all the experiments was from ELGA PURELAB Chorus 1 Complete (Type 1 Water, 18.2 MΩ.cm).

2. Instrumentation and Methods

2.1 Elemental analysis

The contents of carbon, hydrogen and nitrogen were determined using an EA 1110 CHNS CE440 Elemental Analyzer, which belongs to the chemistry microanalysis services in the University of Glasgow.

2.2 Inductively coupled plasma optical emission spectrometry (ICP-OES)

Approximately 5 mg of thoroughly dried sample was weighed exactly and dissolved in 1 mL of HPLC grade water. The sample was covered with a watch glass and heated to boil. Once dissolved, the sample was cooled to room temperature then quantitatively transferred to a clean dry 50.0 mL volumetric flask using HPLC grade water and made up to the mark. A reagent blank was prepared simultaneously under the same conditions. A series of appropriate ICP elemental standards, 1 ppm – 100 ppm, was prepared from commercially available stock solutions. Analysis was performed using an Agilent 5100 SVDV ICP-OES spectrophotometer with peak tracking and Ineloquent enabled.

2.3 Thermogravimetric Analysis (TGA)

Analysis for characterisation was performed on a TA Instruments Q 500 Thermogravimetric analyser under air flow with a heating rate of 10 °C min-1 up to 800 °C. Analysis for Solution Studies were performed on TA Discovery TGA 550 where ~5 mg solid samples were loaded onto a platinum pan, the weight-temperature changes were recorded under N_2 protection.

2.4 FT- IR Spectroscopy

Fourier-transform infrared (FT-IR) spectroscopy: The samples were prepared as a KBr pellet and the FT-IR spectrum was collected in transmission mode in the range of 400-4000 cm^{-1} using a Nicolet IS 50 spectrometer. Wavenumbers are given in cm^{-1}. Intensities are denoted as w = weak, m = medium, s =strong, br = broad, sh = sharp.

2.5 Single Crystal X-Ray Diffraction

Suitable single crystals were selected and mounted onto a rubber loop using Fomblin oil. Single-crystal dataset and unit cells for the compounds were collected at 150(2) K on a Rigaku XtaLAB Synergy R diffractometer equipped with a graphite monochromator (λ (MoKα) = 0.71073 Å) of a micro-focus sealed X-ray source (50 kV, 24.0 mA). Data collection and reduction were performed using the $CrysAlis^{Pro}$

software package and structure solution and refinement were carried out with SHELXT-2019/3[1] and SHELXL-2019[2] via WinGX platform.[3] Most of the non-hydrogen atoms (including those disordered) were anisotropically refined. PLATON SQUEEZE procedure[4] was then applied to calculate the void space, to evaluate the electron count contribution of the remaining solvent water molecules and to produce new HKL files for further structure refinements. Corrections for incident and diffracted beam absorption effects were applied using numerical absorption correction based on Gaussian integration over a multifaceted crystal model.[5] The X-ray crystallographic data in this work have been deposited at the Crystallographic Data Centres with CCDC numbers 2420408-2420411. The data can be obtained free of charge from Cambridge Crystallographic Data Centre service www.ccdc.cam.ac.uk/structures with deposition number.

2.6 pH Measurements

Measurements were taken on a Hanna Instruments HI 9025C microcomputer pH meter, with a BCH combination pH electrode (309-1065) and HI 7669/2W temperature probe.

2.7 Robotic platform

The core robotic hardware shown in Figure S1 consists of a chemical reaction module capable of performing parallel synthesis up to 24 reactors and a heating mantle for 48 reactions (14 mL vials). By using the rotation of the Geneva wheel and high-precision syringe pumps, the chemical reaction module performs liquid handling to achieve highly accurate control of the adding volume of reactant solutions. Na_2MoO_4, L-phenylalanine, hydrazine dihydrochloride and Hydrochloric acid were dissolved in water separately as stock solutions. The adding volumes of these 4 stock solutions are both from 0 to 3.5 mL. After reagents adding, the vials were sealed and transferred manually to the heating mantle, and heat temperature was set up at 65 °C. The reaction was heated and stirred at approximately 65 °C, 500 rpm in a 10 mL disposable glass vial for 2 hours, then left stirring at room temperature for 2 hours. The reaction mixture was left to stand still for 12 hours, remained blue, and then filtered through the high-throughput filtration module and left to crystalize in an open 10mL vial.

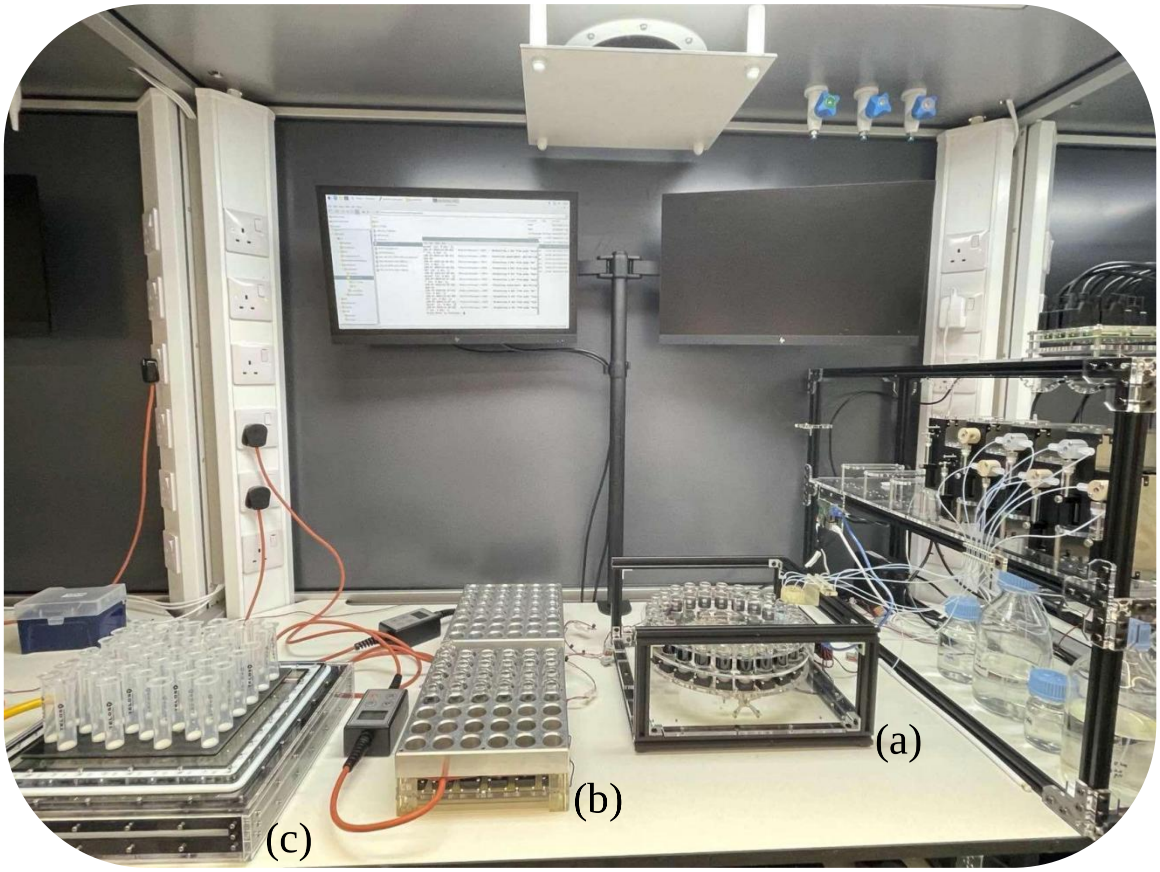

Figure S1: Real image of the platform used for this work: (a) The automatic modular wheel platform (MWP), (b) The heating mantle unit, and (c) The high-throughput filtration module.

3. Programmed Searching Procedures

3.1 Reaction conditions and chemical Spaces 1, 2, 3 and 4

The order of addition, timing and vial size have proven crucial to the formation of the products described in this work. In the case of compounds **1**, **3**, and **4** the persistence of the deep blue solution colour (clear and transparent) is the key to recognizing a successful reaction. All reactions were heated and stirred at approximately 65 °C, 500 rpm in a 10 mL disposable glass vial for 2 hours, then kept naturally cooled down, stirred at room temperature for two hours, left to stand for 12 hours and then filtered through the high-throughput filtration module and left to crystalize in an open 10mL vial. Crystallization times varied from 1 to 2 weeks.

3.1.1 Space 1 stock solutions and reactions:

- Sodium molybdate solution (prepared from $Na_2MoO_4{\cdot}2H_2O$) [0.5 M] adjusted to pH 2.02 using concentrated HCl in H_2O
- L-cysteine in H_2O [0.5 M]
- L-serine in H_2O [0.5 M]
- L-histidine in H_2O [0.5 M]
- L-phenylalanine in H_2O [0.5 M]
- L-tyrosine in H_2O [0.5 M]
- Hydrazine dihydrochloride $N_2H_4{\cdot}2HCl$ in H_2O [0.2 M]
- Hydrochloric acid in H_2O [1.0 M]

Reactions from which the analysed samples of compound **1** was originally found are highlighted in blue. These conditions were repeated many times to determine reproducibility of the compounds.

Space 1 (Search for suitable amino acid ligands for the formation of Mo blues outside the ranges of previously reported reaction conditions) (All values in mL):

Table S1: Reaction conditions for Space 1

Reaction number	Mo	L-Cys	Hydrazine	HCl
Reaction-A1	3.5	1.6	0.5	1.1
Reaction-A2	3.5	1.5	0.5	1.2
Reaction-A3	3.5	1.4	0.5	1.3
Reaction-A4	3.5	1.3	0.5	1.4
Reaction-A5	3.5	1.2	0.5	1.5
Reaction-A6	3.5	1.1	0.5	1.6
Reaction-A7	3.5	1	0.5	1.7
Reaction-A8	3.5	0.9	0.5	1.8
Reaction-A9	3.5	0.8	0.5	1.9

Reaction-A10	3.5	0.7	0.5	1
Reaction-A11	3.5	0.6	0.5	1
Reaction-A12	3.5	0.5	0.5	1
Reaction-A13	3.5	1.6	0.5	1.1
Reaction-A14	3.5	1.5	0.5	1.2
Reaction-A15	3.5	1.4	0.5	1.3
Reaction-A16	3.5	1.3	0.5	1.4
Reaction-A17	3.5	1.2	0.5	1.5
Reaction-A18	3.5	1.1	0.5	1.6
Reaction-A19	3.5	1	0.5	1.7
Reaction-A20	3.5	0.9	0.5	1.8
Reaction-A21	3.5	0.8	0.5	1.9
Reaction-A22	3.5	0.7	0.5	1.1
Reaction-A23	3.5	0.6	0.5	1.2
Reaction-A24	3.5	0.5	0.5	1.3
Reaction-A25	3.5	1.6	1	1.4
Reaction-A26	3.5	1.5	1	0.9
Reaction-A27	3.5	1.4	1	0.8
Reaction-A28	3.5	1.3	1	0.7
Reaction-A29	3.5	1.2	1	0.6
Reaction-A30	3.5	1.1	1	0.5
Reaction-A31	3.5	1	1	1
Reaction-A32	3.5	0.9	1	0.9
Reaction-A33	3.5	0.8	1	0.8
Reaction-A34	3.5	0.7	1	0.7
Reaction-A35	3.5	0.6	1	0.6
Reaction-A36	3.5	0.5	1	0.5
Reaction-A37	3.5	1.6	1	1
Reaction-A38	3.5	1.5	1	0.9
Reaction-A39	3.5	1.4	1	0.8

Reaction-A40	3.5	1.3	1	0.7
Reaction-A41	3.5	1.2	1	0.6
Reaction-A42	3.5	1.1	1	0.5
Reaction-A43	3.5	1	1	1
Reaction-A44	3.5	0.9	1	0.9
Reaction-A45	3.5	0.8	1	0.8
Reaction-A46	3.5	0.7	1	0.7
Reaction-A47	3.5	0.6	1	0.6
Reaction-A48	3.5	0.5	1	0.5
Reaction number	Mo	L-Ser	Hydrazine	HCl
Reaction-A49	3.5	1.6	0.5	1.1
Reaction-A50	3.5	1.5	0.5	1.2
Reaction-A51	3.5	1.4	0.5	1.3
Reaction-A52	3.5	1.3	0.5	1.4
Reaction-A53	3.5	1.2	0.5	1.5
Reaction-A54	3.5	1.1	0.5	1.6
Reaction-A55	3.5	1	0.5	1.7
Reaction-A56	3.5	0.9	0.5	1.8
Reaction-A57	3.5	0.8	0.5	1.9
Reaction-A58	3.5	0.7	0.5	1
Reaction-A59	3.5	0.6	0.5	1
Reaction-A60	3.5	0.5	0.5	1
Reaction-A61	3.5	1.6	0.5	1.1
Reaction-A62	3.5	1.5	0.5	1.2
Reaction-A63	3.5	1.4	0.5	1.3
Reaction-A64	3.5	1.3	0.5	1.4
Reaction-A65	3.5	1.2	0.5	1.5
Reaction-A66	3.5	1.1	0.5	1.6
Reaction-A67	3.5	1	0.5	1.7
Reaction-A68	3.5	0.9	0.5	1.8

Reaction-A69	3.5	0.8	0.5	1.9
Reaction-A70	3.5	0.7	0.5	1.1
Reaction-A71	3.5	0.6	0.5	1.2
Reaction-A72	3.5	0.5	0.5	1.3
Reaction-A73	3.5	1.6	1	1.4
Reaction-A74	3.5	1.5	1	0.9
Reaction-A75	3.5	1.4	1	0.8
Reaction-A76	3.5	1.3	1	0.7
Reaction-A77	3.5	1.2	1	0.6
Reaction-A78	3.5	1.1	1	0.5
Reaction-A79	3.5	1	1	1
Reaction-A80	3.5	0.9	1	0.9
Reaction-A81	3.5	0.8	1	0.8
Reaction-A82	3.5	0.7	1	0.7
Reaction-A83	3.5	0.6	1	0.6
Reaction-A84	3.5	0.5	1	0.5
Reaction-A85	3.5	1.6	1	1
Reaction-A86	3.5	1.5	1	0.9
Reaction-A87	3.5	1.4	1	0.8
Reaction-A88	3.5	1.3	1	0.7
Reaction-A89	3.5	1.2	1	0.6
Reaction-A90	3.5	1.1	1	0.5
Reaction-A91	3.5	1	1	1
Reaction-A92	3.5	0.9	1	0.9
Reaction-A93	3.5	0.8	1	0.8
Reaction-A94	3.5	0.7	1	0.7
Reaction-A95	3.5	0.6	1	0.6
Reaction-A96	3.5	0.5	1	0.5
Reaction number	Mo	L-His	Hydrazine	HCl
Reaction-A97	3.5	1.6	0.5	1.1

Reaction-A98	3.5	1.5	0.5	1.2
Reaction-A99	3.5	1.4	0.5	1.3
Reaction-A100	3.5	1.3	0.5	1.4
Reaction-A101	3.5	1.2	0.5	1.5
Reaction-A102	3.5	1.1	0.5	1.6
Reaction-A103	3.5	1	0.5	1.7
Reaction-A104	3.5	0.9	0.5	1.8
Reaction-A105	3.5	0.8	0.5	1.9
Reaction-A106	3.5	0.7	0.5	1
Reaction-A107	3.5	0.6	0.5	1
Reaction-A108	3.5	0.5	0.5	1
Reaction-A109	3.5	1.6	0.5	1.1
Reaction-A110	3.5	1.5	0.5	1.2
Reaction-A111	3.5	1.4	0.5	1.3
Reaction-A112	3.5	1.3	0.5	1.4
Reaction-A113	3.5	1.2	0.5	1.5
Reaction-A114	3.5	1.1	0.5	1.6
Reaction-A115	3.5	1	0.5	1.7
Reaction-A116	3.5	0.9	0.5	1.8
Reaction-A117	3.5	0.8	0.5	1.9
Reaction-A118	3.5	0.7	0.5	1.1
Reaction-A119	3.5	0.6	0.5	1.2
Reaction-A120	3.5	0.5	0.5	1.3
Reaction-A121	3.5	1.6	1	1.4
Reaction-A122	3.5	1.5	1	0.9
Reaction-A123	3.5	1.4	1	0.8
Reaction-A124	3.5	1.3	1	0.7
Reaction-A125	3.5	1.2	1	0.6
Reaction-A126	3.5	1.1	1	0.5
Reaction-A127	3.5	1	1	1

Reaction-A128	3.5	0.9	1	0.9
Reaction-A129	3.5	0.8	1	0.8
Reaction-A130	3.5	0.7	1	0.7
Reaction-A131	3.5	0.6	1	0.6
Reaction-A132	3.5	0.5	1	0.5
Reaction-A133	3.5	1.6	1	1
Reaction-A134	3.5	1.5	1	0.9
Reaction-A135	3.5	1.4	1	0.8
Reaction-A136	3.5	1.3	1	0.7
Reaction-A137	3.5	1.2	1	0.6
Reaction-A138	3.5	1.1	1	0.5
Reaction-A139	3.5	1	1	1
Reaction-A140	3.5	0.9	1	0.9
Reaction-A141	3.5	0.8	1	0.8
Reaction-A142	3.5	0.7	1	0.7
Reaction-A143	3.5	0.6	1	0.6
Reaction-A144	3.5	0.5	1	0.5
Reaction number	Mo	L-Phe	Hydrazine	HCl
Reaction-A145	3.5	1.6	0.5	1.1
Reaction-A146	3.5	1.5	0.5	1.2
Reaction-A147	3.5	1.4	0.5	1.3
Reaction-A148	3.5	1.3	0.5	1.4
Reaction-A149	3.5	1.2	0.5	1.5
Reaction-A150	3.5	1.1	0.5	1.6
Reaction-A151	3.5	1	0.5	1.7
Reaction-A152	3.5	0.9	0.5	1.8
Reaction-A153	3.5	0.8	0.5	1.9
Reaction-A154	3.5	0.7	0.5	1
Reaction-A155	3.5	0.6	0.5	1
Reaction-A156	3.5	0.5	0.5	1

Reaction-A157	3.5	1.6	0.5	1.1
Reaction-A158	3.5	1.5	0.5	1.2
Reaction-A159	3.5	1.4	0.5	1.3
Reaction-A160	3.5	1.3	0.5	1.4
Reaction-A161	3.5	1.2	0.5	1.5
Reaction-A162	3.5	1.1	0.5	1.6
Reaction-A163	3.5	1	0.5	1.7
Reaction-A164	3.5	0.9	0.5	1.8
Reaction-A165	3.5	0.8	0.5	1.9
Reaction-A166	3.5	0.7	0.5	1.1
Reaction-A167	3.5	0.6	0.5	1.2
Reaction-A168	3.5	0.5	0.5	1.3
Reaction-A169	3.5	1.6	1	1.4
Reaction-A170	3.5	1.5	1	0.9
Reaction-A171	3.5	1.4	1	0.8
Reaction-A172	3.5	1.3	1	0.7
Reaction-A173	3.5	1.2	1	0.6
Reaction-A174	3.5	1.1	1	0.5
Reaction-A175	3.5	1	1	1
Reaction-A176	3.5	0.9	1	0.9
Reaction-A177	3.5	0.8	1	0.8
Reaction-A178	3.5	0.7	1	0.7
Reaction-A179	3.5	0.6	1	0.6
Reaction-A180	3.5	0.5	1	0.5
Reaction-A181	3.5	1.6	1	1
Reaction-A182	3.5	1.5	1	0.9
Reaction-A183	3.5	1.4	1	0.8
Reaction-A184	3.5	1.3	1	0.7
Reaction-A185	3.5	1.2	1	0.6
Reaction-A186	3.5	1.1	1	0.5

Reaction-A187	3.5	1	1	1
Reaction-A188	3.5	0.9	1	0.9
Reaction-A189	3.5	0.8	1	0.8
Reaction-A190	3.5	0.7	1	0.7
Reaction-A191	3.5	0.6	1	0.6
Reaction-A192	3.5	0.5	1	0.5
Reaction number	Mo	L-Tyr	Hydrazine	HCl
Reaction-A193	3.5	1.6	0.5	1.1
Reaction-A194	3.5	1.5	0.5	1.2
Reaction-A195	3.5	1.4	0.5	1.3
Reaction-A196	3.5	1.3	0.5	1.4
Reaction-A197	3.5	1.2	0.5	1.5
Reaction-A198	3.5	1.1	0.5	1.6
Reaction-A199	3.5	1	0.5	1.7
Reaction-A200	3.5	0.9	0.5	1.8
Reaction-A201	3.5	0.8	0.5	1.9
Reaction-A202	3.5	0.7	0.5	1
Reaction-A203	3.5	0.6	0.5	1
Reaction-A204	3.5	0.5	0.5	1
Reaction-A205	3.5	1.6	0.5	1.1
Reaction-A206	3.5	1.5	0.5	1.2
Reaction-A207	3.5	1.4	0.5	1.3
Reaction-A208	3.5	1.3	0.5	1.4
Reaction-A209	3.5	1.2	0.5	1.5
Reaction-A210	3.5	1.1	0.5	1.6
Reaction-A211	3.5	1	0.5	1.7
Reaction-A212	3.5	0.9	0.5	1.8
Reaction-A213	3.5	0.8	0.5	1.9
Reaction-A214	3.5	0.7	0.5	1.1
Reaction-A215	3.5	0.6	0.5	1.2

Reaction-A216	3.5	0.5	0.5	1.3
Reaction-A217	3.5	1.6	1	1.4
Reaction-A218	3.5	1.5	1	0.9
Reaction-A219	3.5	1.4	1	0.8
Reaction-A220	3.5	1.3	1	0.7
Reaction-A221	3.5	1.2	1	0.6
Reaction-A222	3.5	1.1	1	0.5
Reaction-A223	3.5	1	1	1
Reaction-A224	3.5	0.9	1	0.9
Reaction-A225	3.5	0.8	1	0.8
Reaction-A226	3.5	0.7	1	0.7
Reaction-A227	3.5	0.6	1	0.6
Reaction-A228	3.5	0.5	1	0.5
Reaction-A229	3.5	1.6	1	1
Reaction-A230	3.5	1.5	1	0.9
Reaction-A231	3.5	1.4	1	0.8
Reaction-A232	3.5	1.3	1	0.7
Reaction-A233	3.5	1.2	1	0.6
Reaction-A234	3.5	1.1	1	0.5
Reaction-A235	3.5	1	1	1
Reaction-A236	3.5	0.9	1	0.9
Reaction-A237	3.5	0.8	1	0.8
Reaction-A238	3.5	0.7	1	0.7
Reaction-A239	3.5	0.6	1	0.6
Reaction-A240	3.5	0.5	1	0.5

3.1.2 Space 2 (Selection of sodium molybdate concentration to perform optimized reactions）focus on L-phenylalanine ligand

- Sodium molybdate solution [0.2 M]/ [0.5 M]/[1.0 M] adjusted to pH 2.02 using concentrated HCl in H_2O
- L-phenylalanine in H_2O [0.5 M]
- Hydrazine dihydrochloride $N_2H_4 \cdot 2HCl$ in H_2O [0.2 M]
- Hydrochloric acid in H_2O [1.0 M]

Table S2: Reaction conditions for Space 2

Reaction ID	Mo 0.2M	L-Phe	hydrazine	HCl
Reaction_B1	3.5	1	0.5	0.1
Reaction_B2	3.5	1	0.5	0.2
Reaction_B3	3.5	1	0.5	0.3
Reaction_B4	3.5	1	0.5	0.4
Reaction_B5	3.5	1	0.5	0.5
Reaction_B6	3.5	1	0.5	0.6
Reaction_B7	3.5	1	0.5	0.7
Reaction_B8	3.5	1	0.5	0.8
Reaction_B9	3.5	1	0.5	0.9
Reaction_B10	3.5	1	0.5	0.1
Reaction_B11	3.5	1	0.5	0.11
Reaction_B12	3.5	1	0.5	0.12
Reaction_B13	3.5	1	0.5	0.13
Reaction_B14	3.5	1	0.5	0.14
Reaction_B15	3.5	1	0.5	0.15
Reaction_B16	3.5	1	0.5	0.16
Reaction_B17	3.5	1	0.5	0.17
Reaction_B18	3.5	1	0.5	0.18
Reaction_B19	3.5	1	0.5	0.19
Reaction_B20	3.5	1	0.5	0.2
Reaction_B21	3.5	1	0.5	0.21
Reaction_B22	3.5	1	0.5	0.22
Reaction_B23	3.5	1	0.5	0.23

Reaction_B24	3.5	1	0.5	0.24
Reaction_B25	3.5	1	0.5	0.25
Reaction_B26	3.5	1	0.5	0.2
Reaction_B27	3.5	1	0.5	0.3
Reaction_B28	3.5	1	0.5	0.4
Reaction_B29	3.5	1	0.5	0.5
Reaction_B30	3.5	1	0.5	0.6
Reaction_B31	3.5	1	0.5	0.7
Reaction_B32	3.5	1	0.5	0.8
Reaction_B33	3.5	1	0.5	0.9
Reaction_B34	3.5	1	0.5	0.1
Reaction_B35	3.5	1	0.5	0.11
Reaction_B36	3.5	1	0.5	0.12
Reaction_B37	3.5	1	0.5	0.13
Reaction_B38	3.5	1	0.5	0.14
Reaction_B39	3.5	1	0.5	0.15
Reaction_B40	3.5	1	0.5	0.16
Reaction_B41	3.5	1	0.5	0.17
Reaction_B42	3.5	1	0.5	0.18
Reaction_B43	3.5	1	0.5	0.19
Reaction_B44	3.5	1	0.5	0.2
Reaction_B45	3.5	1	0.5	0.21
Reaction_B46	3.5	1	0.5	0.22
Reaction_B47	3.5	1	0.5	0.23
Reaction_B48	3.5	1	0.5	0.24
Reaction ID	Mo 0.5M	L-Phe	hydrazine	HCl
Reaction_B49	3.5	1	0.5	0.1
Reaction_B50	3.5	1	0.5	0.2
Reaction_B51	3.5	1	0.5	0.3
Reaction_B52	3.5	1	0.5	0.4

Reaction_B53	3.5	1	0.5	0.5
Reaction_B54	3.5	1	0.5	0.6
Reaction_B55	3.5	1	0.5	0.7
Reaction_B56	3.5	1	0.5	0.8
Reaction_B57	3.5	1	0.5	0.9
Reaction_B58	3.5	1	0.5	0.1
Reaction_B59	3.5	1	0.5	0.11
Reaction_B60	3.5	1	0.5	0.12
Reaction_B61	3.5	1	0.5	0.13
Reaction_B62	3.5	1	0.5	0.14
Reaction_B63	3.5	1	0.5	0.15
Reaction_B64	3.5	1	0.5	0.16
Reaction_B65	3.5	1	0.5	0.17
Reaction_B66	3.5	1	0.5	0.18
Reaction_B67	3.5	1	0.5	0.19
Reaction_B68	3.5	1	0.5	0.2
Reaction_B69	3.5	1	0.5	0.21
Reaction_B70	3.5	1	0.5	0.22
Reaction_B71	3.5	1	0.5	0.23
Reaction_B72	3.5	1	0.5	0.24
Reaction_B73	3.5	1	0.5	0.25
Reaction_B74	3.5	1	0.5	0.2
Reaction_B75	3.5	1	0.5	0.3
Reaction_B76	3.5	1	0.5	0.4
Reaction_B77	3.5	1	0.5	0.5
Reaction_B78	3.5	1	0.5	0.6
Reaction_B79	3.5	1	0.5	0.7
Reaction_B80	3.5	1	0.5	0.8
Reaction_B81	3.5	1	0.5	0.9
Reaction_B82	3.5	1	0.5	0.1

Reaction_B83	3.5	1	0.5	0.11
Reaction_B84	3.5	1	0.5	0.12
Reaction_B85	3.5	1	0.5	0.13
Reaction_B86	3.5	1	0.5	0.14
Reaction_B87	3.5	1	0.5	0.15
Reaction_B88	3.5	1	0.5	0.16
Reaction_B89	3.5	1	0.5	0.17
Reaction_B90	3.5	1	0.5	0.18
Reaction_B91	3.5	1	0.5	0.19
Reaction_B92	3.5	1	0.5	0.2
Reaction_B93	3.5	1	0.5	0.21
Reaction_B94	3.5	1	0.5	0.22
Reaction_B95	3.5	1	0.5	0.23
Reaction_B96	3.5	1	0.5	0.24
Reaction ID	Mo 1.0M	L-Phe	hydrazine	HCl
Reaction_B97	3.5	1	0.5	0.1
Reaction_B98	3.5	1	0.5	0.2
Reaction_B99	3.5	1	0.5	0.3
Reaction_B100	3.5	1	0.5	0.4
Reaction_B101	3.5	1	0.5	0.5
Reaction_B102	3.5	1	0.5	0.6
Reaction_B103	3.5	1	0.5	0.7
Reaction_B104	3.5	1	0.5	0.8
Reaction_B105	3.5	1	0.5	0.9
Reaction_B106	3.5	1	0.5	0.1
Reaction_B107	3.5	1	0.5	0.11
Reaction_B108	3.5	1	0.5	0.12
Reaction_B109	3.5	1	0.5	0.13
Reaction_B110	3.5	1	0.5	0.14
Reaction_B111	3.5	1	0.5	0.15

Reaction_B112	3.5	1	0.5	0.16
Reaction_B113	3.5	1	0.5	0.17
Reaction_B114	3.5	1	0.5	0.18
Reaction_B115	3.5	1	0.5	0.19
Reaction_B116	3.5	1	0.5	0.2
Reaction_B117	3.5	1	0.5	0.21
Reaction_B118	3.5	1	0.5	0.22
Reaction_B119	3.5	1	0.5	0.23
Reaction_B120	3.5	1	0.5	0.24
Reaction_B121	3.5	1	0.5	0.25
Reaction_B122	3.5	1	0.5	0.2
Reaction_B123	3.5	1	0.5	0.3
Reaction_B124	3.5	1	0.5	0.4
Reaction_B125	3.5	1	0.5	0.5
Reaction_B126	3.5	1	0.5	0.6
Reaction_B127	3.5	1	0.5	0.7
Reaction_B128	3.5	1	0.5	0.8
Reaction_B129	3.5	1	0.5	0.9
Reaction_B130	3.5	1	0.5	0.1
Reaction_B131	3.5	1	0.5	0.11
Reaction_B132	3.5	1	0.5	0.12
Reaction_B133	3.5	1	0.5	0.13
Reaction_B134	3.5	1	0.5	0.14
Reaction_B135	3.5	1	0.5	0.15
Reaction_B136	3.5	1	0.5	0.16
Reaction_B137	3.5	1	0.5	0.17
Reaction_B138	3.5	1	0.5	0.18
Reaction_B139	3.5	1	0.5	0.19
Reaction_B140	3.5	1	0.5	0.2
Reaction_B141	3.5	1	0.5	0.21

Reaction_B142	3.5	1	0.5	0.22
Reaction_B143	3.5	1	0.5	0.23
Reaction_B144	3.5	1	0.5	0.24

The optimization of the robot showed that when sodium molybdate concentration was 0.2 mol/L in the same volume, the highest crystal purity was obtained. The impurities and defects in the crystal are significantly reduced, the crystallinity of the crystal is improved, and the yield is increased (Figure S2).

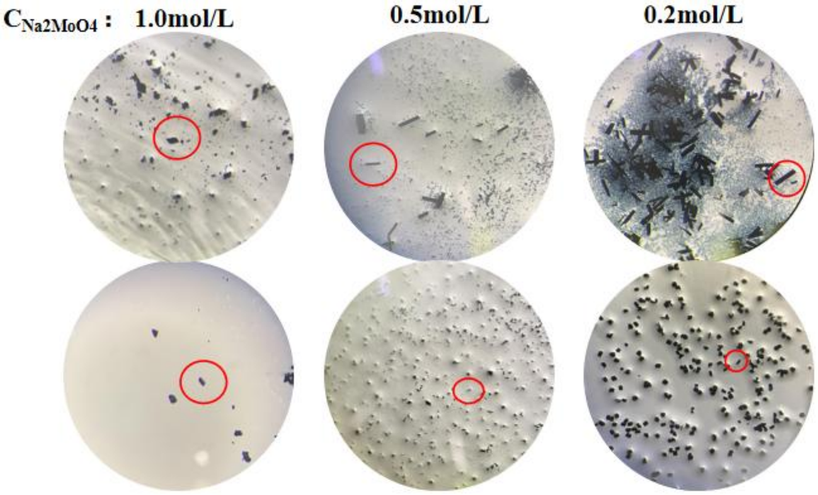

Figure S2. The effect of sodium molybdate concentration on the crystallinity of the product. The highlighted area represents the target optimized columnar crystal, which has the best crystal quality and quantity. Purity of the sample was confirmed by multiple unit cell checks along with structure determination by single crystal x-ray diffraction.

3.1.3 Space 3 (Selection of the HCl for component pH optimization to perform optimized reactions)

- Sodium molybdate dihydrate $Na_2MoO_4 \cdot 2H_2O$ [0.2 M] adjusted to pH 2.02 using conc. HCl in H_2O
- L-phenylalanine in H_2O [0.5 M]
- Hydrazine dihydrochloride $N_2H_4 \cdot 2HCl$ in H_2O [0.2 M]
- Hydrochloric acid in H_2O [1.0 M]

Table S3: Reaction conditions for Space 3

Reaction ID	Mo	L-phe	hydrazine	HCl

Reaction_C_1	3.5	1	0.5	0.1
Reaction_C_2	3.5	1	0.5	0.2
Reaction_C_3	3.5	1	0.5	0.3
Reaction_C_4	3.5	1	0.5	0.4
Reaction_C_5	3.5	1	0.5	0.5
Reaction_C_6	3.5	1	0.5	0.6
Reaction_C_7	3.5	1	0.5	0.7
Reaction_C_8	3.5	1	0.5	0.8
Reaction_C_9	3.5	1	0.5	0.9
Reaction_C_10	3.5	1	0.5	0.1
Reaction_C_11	3.5	1	0.5	0.11
Reaction_C_12	3.5	1	0.5	0.12
Reaction_C_13	3.5	1	0.5	0.13
Reaction_C_14	3.5	1	0.5	0.14
Reaction_C_15	3.5	1	0.5	0.15
Reaction_C_16	3.5	1	0.5	0.16
Reaction_C_17	3.5	1	0.5	0.17
Reaction_C_18	3.5	1	0.5	0.18
Reaction_C_19	3.5	1	0.5	0.19
Reaction_C_20	3.5	1	0.5	0.2
Reaction_C_21	3.5	1	0.5	0.21
Reaction_C_22	3.5	1	0.5	0.22
Reaction_C_23	3.5	1	0.5	0.23
Reaction_C_24	3.5	1	0.5	0.24
Reaction_C_25	3.5	1	0.5	0.25
Reaction_C_26	3.5	1	0.5	0.2
Reaction_C_27	3.5	1	0.5	0.3
Reaction_C_28	3.5	1	0.5	0.4
Reaction_C_29	3.5	1	0.5	0.5
Reaction_C_30	3.5	1	0.5	0.6

Reaction_C_31	3.5	1	0.5	0.7
Reaction_C_32	3.5	1	0.5	0.8
Reaction_C_33	3.5	1	0.5	0.9
Reaction_C_34	3.5	1	0.5	0.1
Reaction_C_35	3.5	1	0.5	0.11
Reaction_C_36	3.5	1	0.5	0.12
Reaction_C_37	3.5	1	0.5	0.13
Reaction_C_38	3.5	1	0.5	0.14
Reaction_C_39	3.5	1	0.5	0.15
Reaction_C_40	3.5	1	0.5	0.16
Reaction_C_41	3.5	1	0.5	0.17
Reaction_C_42	3.5	1	0.5	0.18
Reaction_C_43	3.5	1	0.5	0.19
Reaction_C_44	3.5	1	0.5	0.2
Reaction_C_45	3.5	1	0.5	0.21
Reaction_C_46	3.5	1	0.5	0.22
Reaction_C_47	3.5	1	0.5	0.23
Reaction_C_48	3.5	1	0.5	0.24

pH preferably HCl adjustment, between 0.77-1.02 crystal quality is relatively pure (Fig 3).

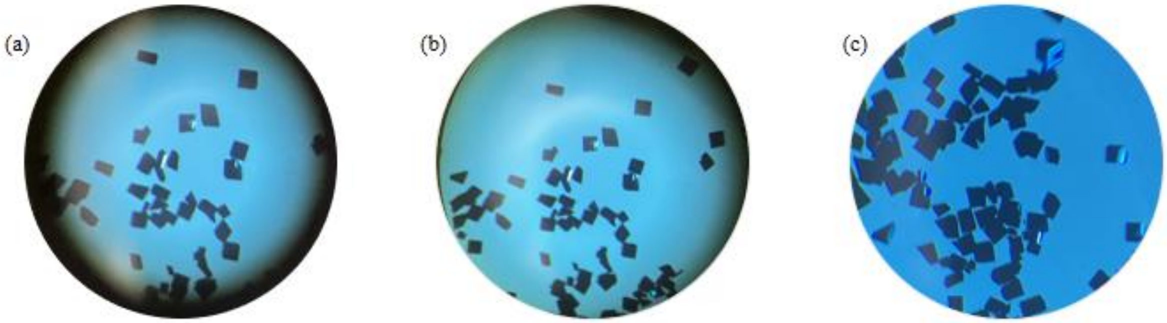

Figure S3. Repeated synthesis of the wheel-like structures of Compound 3 under reaction condition C25 in Table S3. Microscope photos show (a) the first synthesis, (b) the second synthesis and (c) the third synthesis. Purity of the sample was confirmed by multiple unit cell checks along with structure determination by single crystal x-ray diffraction.

3.1.4 Space 4 (Selection of the hydrazine dihydrochloride concentration to perform optimized reactions)

- Sodium molybdate dihydrate $Na_2MoO_4{\cdot}2H_2O$ [0.2 M] adjusted to pH 2.02 using conc. HCl in H_2O
- L-phenylalanine in H_2O [0.5 M]
- Hydrazine dihydrochloride $N_2H_4{\cdot}2HCl$ in H_2O [0.1 M]/ [0.2 M]/[0.3 M]/[0.4 M]/ [0.5 M]/[0.6 M]/ [0.7 M]/[0.8 M]/ [0.9 M]/[1.0 M]
- Hydrochloric acid in H_2O [1.0 M]

Table S4: Reaction conditions for Space 4

Reaction ID	Mo	L-phe	Hydrazine 0.1M	HCl
reaction_D_1	3.5	1	0.5	0.1
reaction_D_2	3.5	1	0.5	0.2
reaction_D_3	3.5	1	0.5	0.3
reaction_D_4	3.5	1	0.5	0.4
reaction_D_5	3.5	1	0.5	0.5
reaction_D_6	3.5	1	0.5	0.6
reaction_D_7	3.5	1	0.5	0.7
reaction_D_8	3.5	1	0.5	0.8
reaction_D_9	3.5	1	0.5	0.9
reaction_D_10	3.5	1	0.5	0.1
reaction_D_11	3.5	1	0.5	0.11
reaction_D_12	3.5	1	0.5	0.12
reaction_D_13	3.5	1	0.5	0.13
reaction_D_14	3.5	1	0.5	0.14
reaction_D_15	3.5	1	0.5	0.15
reaction_D_16	3.5	1	0.5	0.16
reaction_D_17	3.5	1	0.5	0.17
reaction_D_18	3.5	1	0.5	0.18
reaction_D_19	3.5	1	0.5	0.19
reaction_D_20	3.5	1	0.5	0.2
reaction_D_21	3.5	1	0.5	0.21

reaction_D_22	3.5	1	0.5	0.22
reaction_D_23	3.5	1	0.5	0.23
reaction_D_24	3.5	1	0.5	0.24
reaction_D_25	3.5	1	1.0	0.1
reaction_D_26	3.5	1	1.0	0.2
reaction_D_27	3.5	1	1.0	0.3
reaction_D_28	3.5	1	1.0	0.4
reaction_D_29	3.5	1	1.0	0.5
reaction_D_30	3.5	1	1.0	0.6
reaction_D_31	3.5	1	1.0	0.7
reaction_D_32	3.5	1	1.0	0.8
reaction_D_33	3.5	1	1.0	0.9
reaction_D_34	3.5	1	1.0	0.1
reaction_D_35	3.5	1	1.0	0.11
reaction_D_36	3.5	1	1.0	0.12
reaction_D_37	3.5	1	1.0	0.13
reaction_D_38	3.5	1	1.0	0.14
reaction_D_39	3.5	1	1.0	0.15
reaction_D_40	3.5	1	1.0	0.16
reaction_D_41	3.5	1	1.0	0.17
reaction_D_42	3.5	1	1.0	0.18
reaction_D_43	3.5	1	1.0	0.19
reaction_D_44	3.5	1	1.0	0.2
reaction_D_45	3.5	1	1.0	0.21
reaction_D_46	3.5	1	1.0	0.22
reaction_D_47	3.5	1	1.0	0.23
reaction_D_48	3.5	1	1.0	0.24
Reaction ID	Mo	L-phe	Hydrazine 0.2M	HCl
reaction_D_49	3.5	1	0.5	0.1
reaction_D_50	3.5	1	0.5	0.2

reaction_D_51	3.5	1	0.5	0.3
reaction_D_52	3.5	1	0.5	0.4
reaction_D_53	3.5	1	0.5	0.5
reaction_D_54	3.5	1	0.5	0.6
reaction_D_55	3.5	1	0.5	0.7
reaction_D_56	3.5	1	0.5	0.8
reaction_D_57	3.5	1	0.5	0.9
reaction_D_58	3.5	1	0.5	0.1
reaction_D_59	3.5	1	0.5	0.11
reaction_D_60	3.5	1	0.5	0.12
reaction_D_61	3.5	1	0.5	0.13
reaction_D_62	3.5	1	0.5	0.14
reaction_D_63	3.5	1	0.5	0.15
reaction_D_64	3.5	1	0.5	0.16
reaction_D_65	3.5	1	0.5	0.17
reaction_D_66	3.5	1	0.5	0.18
reaction_D_67	3.5	1	0.5	0.19
reaction_D_68	3.5	1	0.5	0.2
reaction_D_69	3.5	1	0.5	0.21
reaction_D_70	3.5	1	0.5	0.22
reaction_D_71	3.5	1	0.5	0.23
reaction_D_72	3.5	1	0.5	0.24
reaction_D_73	3.5	1	1.0	0.1
reaction_D_74	3.5	1	1.0	0.2
reaction_D_75	3.5	1	1.0	0.3
reaction_D_76	3.5	1	1.0	0.4
reaction_D_77	3.5	1	1.0	0.5
reaction_D_78	3.5	1	1.0	0.6
reaction_D_79	3.5	1	1.0	0.7
reaction_D_80	3.5	1	1.0	0.8

reaction_D_81	3.5	1	1.0	0.9
reaction_D_82	3.5	1	1.0	0.1
reaction_D_83	3.5	1	1.0	0.11
reaction_D_84	3.5	1	1.0	0.12
reaction_D_85	3.5	1	1.0	0.13
reaction_D_86	3.5	1	1.0	0.14
reaction_D_87	3.5	1	1.0	0.15
reaction_D_88	3.5	1	1.0	0.16
reaction_D_89	3.5	1	1.0	0.17
reaction_D_90	3.5	1	1.0	0.18
reaction_D_91	3.5	1	1.0	0.19
reaction_D_92	3.5	1	1.0	0.2
reaction_D_93	3.5	1	1.0	0.21
reaction_D_94	3.5	1	1.0	0.22
reaction_D_95	3.5	1	1.0	0.23
reaction_D_96	3.5	1	1.0	0.24
Reaction ID	Mo	L-phe	Hydrazine 0.3M	HCl
reaction_D_97	3.5	1	0.5	0.1
reaction_D_98	3.5	1	0.5	0.2
reaction_D_99	3.5	1	0.5	0.3
reaction_D_100	3.5	1	0.5	0.4
reaction_D_101	3.5	1	0.5	0.5
reaction_D_102	3.5	1	0.5	0.6
reaction_D_103	3.5	1	0.5	0.7
reaction_D_104	3.5	1	0.5	0.8
reaction_D_105	3.5	1	0.5	0.9
reaction_D_106	3.5	1	0.5	0.1
reaction_D_107	3.5	1	0.5	0.11
reaction_D_108	3.5	1	0.5	0.12
reaction_D_109	3.5	1	0.5	0.13

reaction_D_110	3.5	1	0.5	0.14
reaction_D_111	3.5	1	0.5	0.15
reaction_D_112	3.5	1	0.5	0.16
reaction_D_113	3.5	1	0.5	0.17
reaction_D_114	3.5	1	0.5	0.18
reaction_D_115	3.5	1	0.5	0.19
reaction_D_116	3.5	1	0.5	0.2
reaction_D_117	3.5	1	0.5	0.21
reaction_D_118	3.5	1	0.5	0.22
reaction_D_119	3.5	1	0.5	0.23
reaction_D_120	3.5	1	0.5	0.24
reaction_D_121	3.5	1	1.0	0.1
reaction_D_122	3.5	1	1.0	0.2
reaction_D_123	3.5	1	1.0	0.3
reaction_D_124	3.5	1	1.0	0.4
reaction_D_125	3.5	1	1.0	0.5
reaction_D_126	3.5	1	1.0	0.6
reaction_D_127	3.5	1	1.0	0.7
reaction_D_128	3.5	1	1.0	0.8
reaction_D_129	3.5	1	1.0	0.9
reaction_D_130	3.5	1	1.0	0.1
reaction_D_131	3.5	1	1.0	0.11
reaction_D_132	3.5	1	1.0	0.12
reaction_D_133	3.5	1	1.0	0.13
reaction_D_134	3.5	1	1.0	0.14
reaction_D_135	3.5	1	1.0	0.15
reaction_D_136	3.5	1	1.0	0.16
reaction_D_137	3.5	1	1.0	0.17
reaction_D_138	3.5	1	1.0	0.18
reaction_D_139	3.5	1	1.0	0.19

reaction_D_140	3.5	1	1.0	0.2
reaction_D_141	3.5	1	1.0	0.21
reaction_D_142	3.5	1	1.0	0.22
reaction_D_143	3.5	1	1.0	0.23
reaction_D_144	3.5	1	1.0	0.24
Reaction ID	Mo	L-phe	Hydrazine 0.4M	HCl
reaction_D_145	3.5	1	0.5	0.1
reaction_D_146	3.5	1	0.5	0.2
reaction_D_147	3.5	1	0.5	0.3
reaction_D_148	3.5	1	0.5	0.4
reaction_D_149	3.5	1	0.5	0.5
reaction_D_150	3.5	1	0.5	0.6
reaction_D_151	3.5	1	0.5	0.7
reaction_D_152	3.5	1	0.5	0.8
reaction_D_153	3.5	1	0.5	0.9
reaction_D_154	3.5	1	0.5	0.1
reaction_D_155	3.5	1	0.5	0.11
reaction_D_156	3.5	1	0.5	0.12
reaction_D_157	3.5	1	0.5	0.13
reaction_D_158	3.5	1	0.5	0.14
reaction_D_159	3.5	1	0.5	0.15
reaction_D_160	3.5	1	0.5	0.16
reaction_D_161	3.5	1	0.5	0.17
reaction_D_162	3.5	1	0.5	0.18
reaction_D_163	3.5	1	0.5	0.19
reaction_D_164	3.5	1	0.5	0.2
reaction_D_165	3.5	1	0.5	0.21
reaction_D_166	3.5	1	0.5	0.22
reaction_D_167	3.5	1	0.5	0.23
reaction_D_168	3.5	1	0.5	0.24

reaction_D_169	3.5	1	1.0	0.1
reaction_D_170	3.5	1	1.0	0.2
reaction_D_171	3.5	1	1.0	0.3
reaction_D_172	3.5	1	1.0	0.4
reaction_D_173	3.5	1	1.0	0.5
reaction_D_174	3.5	1	1.0	0.6
reaction_D_175	3.5	1	1.0	0.7
reaction_D_176	3.5	1	1.0	0.8
reaction_D_177	3.5	1	1.0	0.9
reaction_D_178	3.5	1	1.0	0.1
reaction_D_179	3.5	1	1.0	0.11
reaction_D_180	3.5	1	1.0	0.12
reaction_D_181	3.5	1	1.0	0.13
reaction_D_182	3.5	1	1.0	0.14
reaction_D_183	3.5	1	1.0	0.15
reaction_D_184	3.5	1	1.0	0.16
reaction_D_185	3.5	1	1.0	0.17
reaction_D_186	3.5	1	1.0	0.18
reaction_D_187	3.5	1	1.0	0.19
reaction_D_188	3.5	1	1.0	0.2
reaction_D_189	3.5	1	1.0	0.21
reaction_D_190	3.5	1	1.0	0.22
reaction_D_191	3.5	1	1.0	0.23
reaction_D_192	3.5	1	1.0	0.24
Reaction ID	Mo	L-phe	Hydrazine 0.5M	HCl
reaction_D_193	3.5	1	0.5	0.1
reaction_D_194	3.5	1	0.5	0.2
reaction_D_195	3.5	1	0.5	0.3
reaction_D_196	3.5	1	0.5	0.4
reaction_D_197	3.5	1	0.5	0.5

reaction_D_198	3.5	1	0.5	0.6
reaction_D_199	3.5	1	0.5	0.7
reaction_D_200	3.5	1	0.5	0.8
reaction_D_201	3.5	1	0.5	0.9
reaction_D_202	3.5	1	0.5	0.1
reaction_D_203	3.5	1	0.5	0.11
reaction_D_204	3.5	1	0.5	0.12
reaction_D_205	3.5	1	0.5	0.13
reaction_D_206	3.5	1	0.5	0.14
reaction_D_207	3.5	1	0.5	0.15
reaction_D_208	3.5	1	0.5	0.16
reaction_D_209	3.5	1	0.5	0.17
reaction_D_210	3.5	1	0.5	0.18
reaction_D_211	3.5	1	0.5	0.19
reaction_D_212	3.5	1	0.5	0.2
reaction_D_213	3.5	1	0.5	0.21
reaction_D_214	3.5	1	0.5	0.22
reaction_D_215	3.5	1	0.5	0.23
reaction_D_216	3.5	1	0.5	0.24
reaction_D_217	3.5	1	1.0	0.1
reaction_D_218	3.5	1	1.0	0.2
reaction_D_219	3.5	1	1.0	0.3
reaction_D_220	3.5	1	1.0	0.4
reaction_D_221	3.5	1	1.0	0.5
reaction_D_222	3.5	1	1.0	0.6
reaction_D_223	3.5	1	1.0	0.7
reaction_D_224	3.5	1	1.0	0.8
reaction_D_225	3.5	1	1.0	0.9
reaction_D_226	3.5	1	1.0	0.1
reaction_D_227	3.5	1	1.0	0.11

reaction_D_228	3.5	1	1.0	0.12
reaction_D_229	3.5	1	1.0	0.13
reaction_D_230	3.5	1	1.0	0.14
reaction_D_231	3.5	1	1.0	0.15
reaction_D_232	3.5	1	1.0	0.16
reaction_D_233	3.5	1	1.0	0.17
reaction_D_234	3.5	1	1.0	0.18
reaction_D_235	3.5	1	1.0	0.19
reaction_D_236	3.5	1	1.0	0.2
reaction_D_237	3.5	1	1.0	0.21
reaction_D_238	3.5	1	1.0	0.22
reaction_D_239	3.5	1	1.0	0.23
reaction_D_240	3.5	1	1.0	0.24
Reaction ID	Mo	L-phe	Hydrazine 0.6M	HCl
reaction_D_241	3.5	1	0.5	0.1
reaction_D_242	3.5	1	0.5	0.2
reaction_D_243	3.5	1	0.5	0.3
reaction_D_244	3.5	1	0.5	0.4
reaction_D_245	3.5	1	0.5	0.5
reaction_D_246	3.5	1	0.5	0.6
reaction_D_247	3.5	1	0.5	0.7
reaction_D_248	3.5	1	0.5	0.8
reaction_D_249	3.5	1	0.5	0.9
reaction_D_250	3.5	1	0.5	0.1
reaction_D_251	3.5	1	0.5	0.11
reaction_D_252	3.5	1	0.5	0.12
reaction_D_253	3.5	1	0.5	0.13
reaction_D_254	3.5	1	0.5	0.14
reaction_D_255	3.5	1	0.5	0.15
reaction_D_256	3.5	1	0.5	0.16

reaction_D_257	3.5	1	0.5	0.17
reaction_D_258	3.5	1	0.5	0.18
reaction_D_259	3.5	1	0.5	0.19
reaction_D_260	3.5	1	0.5	0.2
reaction_D_261	3.5	1	0.5	0.21
reaction_D_262	3.5	1	0.5	0.22
reaction_D_263	3.5	1	0.5	0.23
reaction_D_264	3.5	1	0.5	0.24
reaction_D_265	3.5	1	1.0	0.1
reaction_D_266	3.5	1	1.0	0.2
reaction_D_267	3.5	1	1.0	0.3
reaction_D_268	3.5	1	1.0	0.4
reaction_D_269	3.5	1	1.0	0.5
reaction_D_270	3.5	1	1.0	0.6
reaction_D_271	3.5	1	1.0	0.7
reaction_D_272	3.5	1	1.0	0.8
reaction_D_273	3.5	1	1.0	0.9
reaction_D_274	3.5	1	1.0	0.1
reaction_D_275	3.5	1	1.0	0.11
reaction_D_276	3.5	1	1.0	0.12
reaction_D_277	3.5	1	1.0	0.13
reaction_D_278	3.5	1	1.0	0.14
reaction_D_279	3.5	1	1.0	0.15
reaction_D_280	3.5	1	1.0	0.16
reaction_D_281	3.5	1	1.0	0.17
reaction_D_282	3.5	1	1.0	0.18
reaction_D_283	3.5	1	1.0	0.19
reaction_D_284	3.5	1	1.0	0.2
reaction_D_285	3.5	1	1.0	0.21
reaction_D_286	3.5	1	1.0	0.22

reaction_D_287	3.5	1	1.0	0.23
reaction_D_288	3.5	1	1.0	0.24
Reaction ID	Mo	L-phe	Hydrazine 0.7M	HCl
reaction_D_289	3.5	1	0.5	0.1
reaction_D_290	3.5	1	0.5	0.2
reaction_D_291	3.5	1	0.5	0.3
reaction_D_292	3.5	1	0.5	0.4
reaction_D_293	3.5	1	0.5	0.5
reaction_D_294	3.5	1	0.5	0.6
reaction_D_295	3.5	1	0.5	0.7
reaction_D_296	3.5	1	0.5	0.8
reaction_D_297	3.5	1	0.5	0.9
reaction_D_298	3.5	1	0.5	0.1
reaction_D_299	3.5	1	0.5	0.11
reaction_D_300	3.5	1	0.5	0.12
reaction_D_301	3.5	1	0.5	0.13
reaction_D_302	3.5	1	0.5	0.14
reaction_D_303	3.5	1	0.5	0.15
reaction_D_304	3.5	1	0.5	0.16
reaction_D_305	3.5	1	0.5	0.17
reaction_D_306	3.5	1	0.5	0.18
reaction_D_307	3.5	1	0.5	0.19
reaction_D_308	3.5	1	0.5	0.2
reaction_D_309	3.5	1	0.5	0.21
reaction_D_310	3.5	1	0.5	0.22
reaction_D_311	3.5	1	0.5	0.23
reaction_D_312	3.5	1	0.5	0.24
reaction_D_313	3.5	1	1.0	0.1
reaction_D_314	3.5	1	1.0	0.2
reaction_D_315	3.5	1	1.0	0.3

reaction_D_316	3.5	1	1.0	0.4
reaction_D_317	3.5	1	1.0	0.5
reaction_D_318	3.5	1	1.0	0.6
reaction_D_319	3.5	1	1.0	0.7
reaction_D_320	3.5	1	1.0	0.8
reaction_D_321	3.5	1	1.0	0.9
reaction_D_322	3.5	1	1.0	0.1
reaction_D_323	3.5	1	1.0	0.11
reaction_D_324	3.5	1	1.0	0.12
reaction_D_325	3.5	1	1.0	0.13
reaction_D_326	3.5	1	1.0	0.14
reaction_D_327	3.5	1	1.0	0.15
reaction_D_328	3.5	1	1.0	0.16
reaction_D_329	3.5	1	1.0	0.17
reaction_D_330	3.5	1	1.0	0.18
reaction_D_331	3.5	1	1.0	0.19
reaction_D_332	3.5	1	1.0	0.2
reaction_D_333	3.5	1	1.0	0.21
reaction_D_334	3.5	1	1.0	0.22
reaction_D_335	3.5	1	1.0	0.23
reaction_D_336	3.5	1	1.0	0.24
Reaction ID	Mo	L-phe	Hydrazine 0.8M	HCl
reaction_D_337	3.5	1	0.5	0.1
reaction_D_338	3.5	1	0.5	0.2
reaction_D_339	3.5	1	0.5	0.3
reaction_D_340	3.5	1	0.5	0.4
reaction_D_341	3.5	1	0.5	0.5
reaction_D_342	3.5	1	0.5	0.6
reaction_D_343	3.5	1	0.5	0.7
reaction_D_344	3.5	1	0.5	0.8

reaction_D_345	3.5	1	0.5	0.9
reaction_D_346	3.5	1	0.5	0.1
reaction_D_347	3.5	1	0.5	0.11
reaction_D_348	3.5	1	0.5	0.12
reaction_D_349	3.5	1	0.5	0.13
reaction_D_350	3.5	1	0.5	0.14
reaction_D_351	3.5	1	0.5	0.15
reaction_D_352	3.5	1	0.5	0.16
reaction_D_353	3.5	1	0.5	0.17
reaction_D_354	3.5	1	0.5	0.18
reaction_D_355	3.5	1	0.5	0.19
reaction_D_356	3.5	1	0.5	0.2
reaction_D_357	3.5	1	0.5	0.21
reaction_D_358	3.5	1	0.5	0.22
reaction_D_359	3.5	1	0.5	0.23
reaction_D_360	3.5	1	0.5	0.24
reaction_D_361	3.5	1	1.0	0.1
reaction_D_362	3.5	1	1.0	0.2
reaction_D_363	3.5	1	1.0	0.3
reaction_D_364	3.5	1	1.0	0.4
reaction_D_365	3.5	1	1.0	0.5
reaction_D_366	3.5	1	1.0	0.6
reaction_D_367	3.5	1	1.0	0.7
reaction_D_368	3.5	1	1.0	0.8
reaction_D_369	3.5	1	1.0	0.9
reaction_D_370	3.5	1	1.0	0.1
reaction_D_371	3.5	1	1.0	0.11
reaction_D_372	3.5	1	1.0	0.12
reaction_D_373	3.5	1	1.0	0.13
reaction_D_374	3.5	1	1.0	0.14

reaction_D_375	3.5	1	1.0	0.15
reaction_D_376	3.5	1	1.0	0.16
reaction_D_377	3.5	1	1.0	0.17
reaction_D_378	3.5	1	1.0	0.18
reaction_D_379	3.5	1	1.0	0.19
reaction_D_380	3.5	1	1.0	0.2
reaction_D_381	3.5	1	1.0	0.21
reaction_D_382	3.5	1	1.0	0.22
reaction_D_383	3.5	1	1.0	0.23
reaction_D_384	3.5	1	1.0	0.24
Reaction ID	Mo	L-phe	Hydrazine 0.9M	HCl
reaction_D_385	3.5	1	0.5	0.1
reaction_D_386	3.5	1	0.5	0.2
reaction_D_387	3.5	1	0.5	0.3
reaction_D_388	3.5	1	0.5	0.4
reaction_D_389	3.5	1	0.5	0.5
reaction_D_390	3.5	1	0.5	0.6
reaction_D_391	3.5	1	0.5	0.7
reaction_D_392	3.5	1	0.5	0.8
reaction_D_393	3.5	1	0.5	0.9
reaction_D_394	3.5	1	0.5	0.1
reaction_D_395	3.5	1	0.5	0.11
reaction_D_396	3.5	1	0.5	0.12
reaction_D_397	3.5	1	0.5	0.13
reaction_D_398	3.5	1	0.5	0.14
reaction_D_399	3.5	1	0.5	0.15
reaction_D_400	3.5	1	0.5	0.16
reaction_D_401	3.5	1	0.5	0.17
reaction_D_402	3.5	1	0.5	0.18
reaction_D_403	3.5	1	0.5	0.19

reaction_D_404	3.5	1	0.5	0.2
reaction_D_405	3.5	1	0.5	0.21
reaction_D_406	3.5	1	0.5	0.22
reaction_D_407	3.5	1	0.5	0.23
reaction_D_408	3.5	1	0.5	0.24
reaction_D_409	3.5	1	1.0	0.1
reaction_D_410	3.5	1	1.0	0.2
reaction_D_411	3.5	1	1.0	0.3
reaction_D_412	3.5	1	1.0	0.4
reaction_D_413	3.5	1	1.0	0.5
reaction_D_414	3.5	1	1.0	0.6
reaction_D_415	3.5	1	1.0	0.7
reaction_D_416	3.5	1	1.0	0.8
reaction_D_417	3.5	1	1.0	0.9
reaction_D_418	3.5	1	1.0	0.1
reaction_D_419	3.5	1	1.0	0.11
reaction_D_420	3.5	1	1.0	0.12
reaction_D_421	3.5	1	1.0	0.13
reaction_D_422	3.5	1	1.0	0.14
reaction_D_423	3.5	1	1.0	0.15
reaction_D_424	3.5	1	1.0	0.16
reaction_D_425	3.5	1	1.0	0.17
reaction_D_426	3.5	1	1.0	0.18
reaction_D_427	3.5	1	1.0	0.19
reaction_D_428	3.5	1	1.0	0.2
reaction_D_429	3.5	1	1.0	0.21
reaction_D_430	3.5	1	1.0	0.22
reaction_D_431	3.5	1	1.0	0.23
reaction_D_432	3.5	1	1.0	0.24
Reaction ID	Mo	L-phe	Hydrazine 0.9M	HCl

reaction_D_433	3.5	1	0.5	0.1
reaction_D_434	3.5	1	0.5	0.2
reaction_D_435	3.5	1	0.5	0.3
reaction_D_436	3.5	1	0.5	0.4
reaction_D_437	3.5	1	0.5	0.5
reaction_D_438	3.5	1	0.5	0.6
reaction_D_439	3.5	1	0.5	0.7
reaction_D_440	3.5	1	0.5	0.8
reaction_D_441	3.5	1	0.5	0.9
reaction_D_442	3.5	1	0.5	0.1
reaction_D_443	3.5	1	0.5	0.11
reaction_D_444	3.5	1	0.5	0.12
reaction_D_445	3.5	1	0.5	0.13
reaction_D_446	3.5	1	0.5	0.14
reaction_D_447	3.5	1	0.5	0.15
reaction_D_448	3.5	1	0.5	0.16
reaction_D_449	3.5	1	0.5	0.17
reaction_D_450	3.5	1	0.5	0.18
reaction_D_451	3.5	1	0.5	0.19
reaction_D_452	3.5	1	0.5	0.2
reaction_D_453	3.5	1	0.5	0.21
reaction_D_454	3.5	1	0.5	0.22
reaction_D_455	3.5	1	0.5	0.23
reaction_D_456	3.5	1	0.5	0.24
reaction_D_457	3.5	1	1.0	0.1
reaction_D_458	3.5	1	1.0	0.2
reaction_D_459	3.5	1	1.0	0.3
reaction_D_460	3.5	1	1.0	0.4
reaction_D_461	3.5	1	1.0	0.5
reaction_D_462	3.5	1	1.0	0.6

reaction_D_463	3.5	1	1.0	0.7
reaction_D_464	3.5	1	1.0	0.8
reaction_D_465	3.5	1	1.0	0.9
reaction_D_466	3.5	1	1.0	0.1
reaction_D_467	3.5	1	1.0	0.11
reaction_D_468	3.5	1	1.0	0.12
reaction_D_469	3.5	1	1.0	0.13
reaction_D_470	3.5	1	1.0	0.14
reaction_D_471	3.5	1	1.0	0.15
reaction_D_472	3.5	1	1.0	0.16
reaction_D_473	3.5	1	1.0	0.17
reaction_D_474	3.5	1	1.0	0.18
reaction_D_475	3.5	1	1.0	0.19
reaction_D_476	3.5	1	1.0	0.2
reaction_D_477	3.5	1	1.0	0.21
reaction_D_478	3.5	1	1.0	0.22
reaction_D_479	3.5	1	1.0	0.23
reaction_D_480	3.5	1	1.0	0.24

3.2 Reaction conditions and chemical Space 5

The order of addition, timing and vial size has proven to be crucial to the formation of the products described in this work. In the case of compound **2** the persistence of the deep blue solution colour (clear and transparent) is the key to recognizing a successful reaction. All reactions were stirred at 500 rpm in a 10 mL disposable glass vial for 12 hours at room temperature. Crystallization times varied about 8 days.

3.2.1 Space 5 (The optimized reactions)

- Sodium molybdate dihydrate $Na_2MoO_4{\cdot}2H_2O$ [0.2 M] adjusted to pH 2.02 using conc. HCl in H_2O
- L-phenylalanine in H_2O [0.5 M]
- Hydrazine dihydrochloride $N_2H_4{\cdot}2HCl$ in H_2O [0.5 M]
- Hydrochloric acid in H_2O [1.0 M]

Table S5: Reaction conditions for Space 5

Reaction ID	Mo	L-Phe	hydrazine	HCl
Reaction_O1	3.5	1	0.5	0.1
Reaction_O2	3.5	1	0.5	0.2
Reaction_O3	3.5	1	0.5	0.3
Reaction_O4	3.5	1	0.5	0.4
Reaction_O5	3.5	1	0.5	0.5
Reaction_O6	3.5	1	0.5	0.6
Reaction_O7	3.5	1	0.5	0.7
Reaction_O8	3.5	1	0.5	0.8
Reaction_O9	3.5	1	0.5	0.9
Reaction_O10	3.5	1	0.5	0.1
Reaction_O11	3.5	1	0.5	0.11
Reaction_O12	3.5	1	0.5	0.12
Reaction_O13	3.5	1	0.5	0.13
Reaction_O14	3.5	1	0.5	0.14
Reaction_O15	3.5	1	0.5	0.15
Reaction_O16	3.5	1	0.5	0.16
Reaction_O17	3.5	1	0.5	0.17
Reaction_O18	3.5	1	0.5	0.18
Reaction_O19	3.5	1	0.5	0.19
Reaction_O20	3.5	1	0.5	0.2
Reaction_O21	3.5	1	0.5	0.21
Reaction_O22	3.5	1	0.5	0.22
Reaction_O23	3.5	1	0.5	0.23
Reaction_O24	3.5	1	0.5	0.24
Reaction_O25	3.5	1	0.5	0.25
Reaction_O26	3.5	1	0.5	0.2
Reaction_O27	3.5	1	0.5	0.3
Reaction_O28	3.5	1	0.5	0.4
Reaction_O29	3.5	1	0.5	0.5

Reaction_O30	3.5	1	0.5	0.6
Reaction_O31	3.5	1	0.5	0.7
Reaction_O32	3.5	1	0.5	0.8
Reaction_O33	3.5	1	0.5	0.9
Reaction_O34	3.5	1	0.5	0.1
Reaction_O35	3.5	1	0.5	0.11
Reaction_O36	3.5	1	0.5	0.12
Reaction_O37	3.5	1	0.5	0.13
Reaction_O38	3.5	1	0.5	0.14
Reaction_O39	3.5	1	0.5	0.15
Reaction_O40	3.5	1	0.5	0.16
Reaction_O41	3.5	1	0.5	0.17
Reaction_O42	3.5	1	0.5	0.18
Reaction_O43	3.5	1	0.5	0.19
Reaction_O44	3.5	1	0.5	0.2
Reaction_O45	3.5	1	0.5	0.21
Reaction_O46	3.5	1	0.5	0.22
Reaction_O47	3.5	1	0.5	0.23
Reaction_O48	3.5	1	0.5	0.24

4. Optimised Synthetic Procedures

4.1 Compound 1 {Mo_{154}(L-Phe)$_{10}$} – Initial Discovery

$Na_2MoO_4 \cdot 2H_2O$ (300.0 mg, 1.2 mmol) in water (35 mL). The solution was acidified to pH 2.10 by addition of 1 M HCl (25 ml). L-Phenylalanine (375.0 mg, 2.3 mmol) and 0.2 M hydrazine dihydrochloride (1.5 ml) were added to the solution. 1 M HCl was added dropwise until the pH of the solution was 1.85, and the mixture was heated and stirred in a 100 mL Erlenmeyer flask at 85 °C for 2 h. After stirred at room temperature for further two hours and standing for 24 hours, the solution was filtered and the resulting clear dark blue solution was stored for crystallisation for three weeks. The resulting dark blue bulk crystals were collected by filtration.

4.2 Compound 1 {Mo_{154}(L-Phe)$_{10}$} – Optimal Reactant

The reactants compound **1** were dispensed in immediate succession in the order below:

- Sodium molybdate dihydrate $Na_2MoO_4 \cdot 2H_2O$ [0.5 M] adjusted to pH 2.02 using conc HCl in H_2O

- L-phenylalanine in H_2O [0.5 M]
- Hydrazine dihydrochloride in H_2O $N_2H_4·2HCl$ [0.2 M]
- Hydrochloric acid in H_2O HCl [1.0 M]

Under stirring aqueous sodium molybdate (3.5 mL, $Na_2MoO_4·2H_2O$, [0.5 M], pH 2.02) stock solution was dispensed into a 10 mL vial. Aqueous L-phenylalanine (1.2 mL, $C_9H_{11}NO_2$ [0.5 M]) was added. Aqueous hydrazine dihydrochloride (1.0 mL, $N_2H_4·2HCl$ [0.2 M]) was added, colour change from clear to deep blue occurred upon addition. Hydrochloric acid [0.6 mL, 1.0 M] added. The reaction was heated and stirred at approximately 65 °C, 500 rpm in a 10 mL disposable glass vial for 2 hours, then left stirring at room temperature for 2 hour. The reaction mixture was left to stand still for 12 hours, remained blue, and then filtered through the high-throughput filtration module and left to crystalize in an open 10mL vial. After 1 week black crystals were obtained (SI section 3.1.1 Table S1. A173).

4.3 Compound 2 {Mo_{154}(L-Phe)$_9$}

The reactants for compound **2** were dispensed in immediate succession in the order below:

- Sodium molybdate dihydrate $Na_2MoO_4·2H_2O$ [0.2M] adjusted to pH 2.02 using conc HCl in H_2O
- L-phenylalanine in H_2O [0.5 M]
- Hydrazine dihydrochloride $N_2H_4·2HCl$ in H_2O [0.5 M]
- Hydrochloric acid HCl in H_2O [1.0 M]

Under stirring aqueous sodium molybdate (3.5 mL, $Na_2MoO_4.2H_2O$, [0.2 M], pH 2.02) stock solution was dispensed into a 10 mL vial. Aqueous L-phenylalanine (1.0 mL, $C_9H_{11}NO_2$ [0.5 M]) was added. Aqueous hydrazine dihydrochloride (0.5 mL, $N_2H_4·2HCl$ [0.5 M]) was added, colour change from clear to deep blue occurred upon addition. Hydrochloric acid [0.8 mL, 1.0 M] added. The reaction was stirred at 500 rpm in a 10 mL disposable glass vial at room temperature for 12 hours. The reaction mixture was left to stand for 12 hours, remained clear blue. After 8 days black crystals were obtained (SI section 3.2.1 Table S5. O8 and O32).

4.4 Compound 3 1D {Mo_{154}(L-Phe)$_7$}

The reactants for compound **3** were dispensed in immediate succession in the order below:

- Sodium molybdate dihydrate $Na_2MoO_4·2H_2O$ [0.2M] adjusted to pH 2.02 using conc HCl in H_2O
- L-phenylalanine in H_2O [0.5 M]
- Hydrazine dihydrochloride $N_2H_4·2HCl$ in H_2O [0.2 M] in H_2O
- Hydrochloric acid HCl in H_2O [1.0 M] in H_2O

Under stirring aqueous sodium molybdate (3.5 mL, $Na_2MoO_4.2H_2O$, [0.2 M], pH 2.02) stock solution was dispensed into a 10 mL vial. Aqueous L-phenylalanine (1.0 mL, $C_9H_{11}NO_2$ [0.5 M]) was added. Aqueous hydrazine dihydrochloride (0.5 mL, $N_2H_4·2HCl$ [0.2 M]) was added, colour change from clear to deep blue occurred upon addition. Hydrochloric acid [0.25 mL, 1.0 M] was added. The reaction was heated and stirred at approximately 65 °C, 500 rpm in a 10 mL disposable glass vial for 2 hours, then left stirring at room temperature for 2 hours. The reaction mixture was left to stand for 12 hours, remained blue, and then filtered through the high-throughput filtration module and left to crystalize in an open 10mL vial. After 1 week black crystals were obtained (SI section 3.1.3 Table S3. C25).

4.5 Compound 4 2D {Mo_{154}(L-Phe)$_6$}

The reactants for compound **4** were dispensed in immediate succession in the order below:

- Sodium molybdate dihydrate $Na_2MoO_4 \cdot 2H_2O$ [0.2M] adjusted to pH 2.02 using conc HCl in H_2O
- L-phenylalanine in H_2O [0.5 M]
- Hydrazine dihydrochloride $N_2H_4 \cdot 2HCl$ in H_2O [0.5 M]
- Hydrochloric acid HCl in H_2O [1.0 M]

Under stirring aqueous sodium molybdate (3.5 mL, $Na_2MoO_4.2H_2O$, [0.2 M], pH 2.02) stock solution was dispensed into a 10 mL vial. Aqueous L-phenylalanine (1.0 mL, $C_9H_{11}NO_2$ [0.5 M]) was added. Aqueous hydrazine dihydrochloride (0.5 mL, $N_2H_4 \cdot 2HCl$ [0.5 M]) was added, colour change from clear to deep blue occurred upon addition. Hydrochloric acid [0.21 mL, 1.0 M] was added. The reaction was heated and stirred at approximately 65 °C, 500 rpm in a 10 mL disposable glass vial for 2 hours, then left stirring at room temperature for 2 hours. The reaction mixture was left to stand for 12 hours, remained blue, and then filtered through the high-throughput filtration module and left to crystalize in an open 10mL vial. After 4 days black crystals were obtained (SI section 3.1.4 Table S4. D213).

5. Crystallographic Data

Table S6. Crystal data and structure refinement details for $Na_6H_8[Mo_{154}H_{14}O_{462}(H_2O)_{50}(C_9H_{11}NO_2)_{10}]\cdot 180H_2O$ **1**.

Identification code	**1**	
Empirical formula	$C_{90}H_{592}Mo_{154}N_{10}Na_6O_{712}$	
Formula weight	28122.40	
Temperature	150(2) K	
Wavelength	0.71073 Å	
Crystal system	Monoclinic	
Space group	*C*2	
Unit cell dimensions	a = 36.4929(3) Å	α = 90°.
	b = 42.8827(2) Å	β = 108.5250(10)°.
	c = 31.3674(2) Å	γ = 90°.
Volume	46543.8(6) $Å^3$	
Z	2	
Density (calculated)	2.007 Mg/m^3	
Absorption coefficient	2.095 mm^{-1}	
F(000)	26864	
Crystal size	0.800 x 0.112 x 0.032 mm^3	
Theta range for data collection	2.26 to 26.00°.	
Index ranges	-45<=h<=45, -52<=k<=52, -38<=l<=38	
Reflections collected	444297	
Independent reflections	91390 [R(int) = 0.0504]	
Completeness to theta = 25.242°	99.9 %	
Absorption correction	Gaussian	
Max. and min. transmission	1.000 and 0.841	
Refinement method	Full-matrix least-squares on F^2	
Data / restraints / parameters	91390 / 91 / 3840	
Goodness-of-fit on F^2	1.033	
Final R indices [I>2sigma(I)]	R1 = 0.0377, wR2 = 0.1015	
R indices (all data)	R1 = 0.0508, wR2 = 0.1077	
Absolute structure parameter	0.12(3)	
Largest diff. peak and hole	1.51 and -0.64 e.$Å^{-3}$	

Table S7. Crystal data and structure refinement for $Na_4H_{10}[Mo_{154}H_{14}O_{462}(H_2O)_{52}(C_9H_{11}NO_2)_9]\cdot 230H_2O$ **2**.

Identification code	**2**	
Empirical formula	$C_{81}H_{687}Mo_{154}N_9Na_4O_{762}$	
Formula weight	28850.08	
Temperature	150(2) K	
Wavelength	0.71073 Å	
Crystal system	Orthorhombic	
Space group	$P2_12_12$	
Unit cell dimensions	a = 46.7285(2) Å	α= 90°.
	b = 44.1396(3) Å	β= 90°.
	c = 47.1586(2) Å	γ = 90°.
Volume	97268.2(9) $Å^3$	
Z	4	
Density (calculated)	1.970 Mg/m^3	
Absorption coefficient	2.010 mm^{-1}	
F(000)	55376	
Crystal size	0.220 x 0.120 x 0.070 mm^3	
Theta range for data collection	2.222 to 26.000°.	
Index ranges	-56<=h<=57, -53<=k<=53, -57<=l<=57	
Reflections collected	2078737	
Independent reflections	187106 [R(int) = 0.1411]	
Completeness to theta = 25.242°	99.9 %	
Absorption correction	Gaussian	
Max. and min. transmission	1.000 and 0.477	
Refinement method	Full-matrix least-squares on F^2	
Data / restraints / parameters	187106 / 416 / 6831	
Goodness-of-fit on F^2	1.046	
Final R indices [I>2sigma(I)]	R1 = 0.0521, wR2 = 0.1274	
R indices (all data)	R1 = 0.0802, wR2 = 0.1505	
Absolute structure parameter	0.10(3)	
Largest diff. peak and hole	1.40 and -0.95 e.$Å^{-3}$	

Table S8. Crystal data and structure refinement for $Na_{12}H_{16}[Mo_{154}O_{462}H_{14}(H_2O)_{56}(C_9H_{11}NO_2)_7][Mo_{154}O_{462}H_{14}(H_2O)_{51}(C_9H_{11}NO_2)_7]\cdot 460H_2O$ **3**.

Identification code	3	
Empirical formula	$C_{126}H_{1332}Mo_{308}N_{14}Na_{12}O_{1519}$	
Formula weight	57181.38	
Temperature	150(2) K	
Wavelength	0.71073 Å	
Crystal system	Triclinic	
Space group	*P* -1	
Unit cell dimensions	a = 30.4172(2) Å	α= 93.9010(10)°.
	b = 34.0113(2) Å	β= 94.7110(10)°.
	c = 46.8336(2) Å	γ = 95.1660(10)°.
Volume	47951.8(5) $Å^3$	
Z	1	
Density (calculated)	1.980 Mg/m^3	
Absorption coefficient	2.038 mm^{-1}	
F(000)	27406	
Crystal size	0.136 x 0.105 x 0.020 mm^3	
Theta range for data collection	2.200 to 25.068°.	
Index ranges	-36<=h<=36, -40<=k<=40, -55<=l<=55	
Reflections collected	1816573	
Independent reflections	169615 [R(int) = 0.0661]	
Completeness to theta = 25.068°	99.7 %	
Absorption correction	Gaussian	
Max. and min. transmission	1.000 and 0.691	
Refinement method	Full-matrix least-squares on F^2	
Data / restraints / parameters	169615 / 310 / 6438	
Goodness-of-fit on F^2	1.025	
Final R indices [I>2sigma(I)]	R1 = 0.0509, wR2 = 0.1427	
R indices (all data)	R1 = 0.0722, wR2 = 0.1578	
Largest diff. peak and hole	2.10 and -1.89 e.$Å^{-3}$	

Table S9. Crystal data and structure refinement for $Na_{11}H_3[Mo_{154}H_{14}O_{462}(H_2O)_{56}(C_9H_{11}NO_2)_6]\cdot 200H_2O$ **4**.

Identification code	**4**	
Empirical formula	$C_{54}H_{595}Mo_{154}N_6Na_{11}O_{730}$	
Formula weight	28039.98	
Temperature	150(2) K	
Wavelength	0.71073 Å	
Crystal system	Orthorhombic	
Space group	*Aba*2	
Unit cell dimensions	a = 30.38740(10) Å	α = 90°.
	b = 56.1686(2) Å	β = 90°.
	c = 50.1012(2) Å	γ = 90°.
Volume	85513.6(5) $Å^3$	
Z	4	
Density (calculated)	2.178 Mg/m^3	
Absorption coefficient	2.283 mm^{-1}	
F(000)	53560	
Crystal size	0.110 x 0.070 x 0.020 mm^3	
Theta range for data collection	2.276 to 26.000°.	
Index ranges	-37<=h<=37, -68<=k<=69, -61<=l<=61	
Reflections collected	810481	
Independent reflections	83987 [R(int) = 0.0413]	
Completeness to theta = 25.242°	99.9 %	
Absorption correction	Gaussian	
Max. and min. transmission	1.000 and 0.747	
Refinement method	Full-matrix least-squares on F^2	
Data / restraints / parameters	83987 / 652 / 3854	
Goodness-of-fit on F^2	1.031	
Final R indices [I>2sigma(I)]	R1 = 0.0309, wR2 = 0.0823	
R indices (all data)	R1 = 0.0384, wR2 = 0.0862	
Absolute structure parameter	0.5	
Largest diff. peak and hole	1.47 and -1.59 e.$Å^{-3}$	

6. Structure Analysis and Description

Table S10. Brief summary of crystal data for comparison of lattices, unit cells and volumes per cluster†

	Struct type	Crystal Lattice	Space Group	Z	Unit cell parameters							Vol per cluster (Å^3)
					a (Å)	b (Å)	c (Å)	α (°)	β (°)	γ (°)	Vol (Å^3)	
**1**	0-D	Monoclinic	*C*2	2	36.49	42.88	31.36	90	108.5	90	46543	23271
**2**	0-D	Orthorhombic	$P2_12_12$	4	46.73	44.14	47.15	90	90	90	97268	24137
**3**	1-D	Triclinic	*P*-1	2	30.42	34.01	46.83	93.9	94.7	95.2	47952	23976
**4**	2-D	Orthorhombic	*Aea*2	4	30.38	56.17	50.10	90	90	90	85513	21378

† Volume per cluster (VPC) is defined by the volume that each cluster with associated cation and anion and solvent molecules averagely occupies in solid state single crystal structure. It is calculated from the unit cell volume divided by number of the clusters (Z) in the unit cell.

All four structures are found to be {Mo_{154}} type Mo blue with phenylalanine as ligands coordinating to {Mo_2} dimers inside the {Mo_{154}} clusters. Each structure has nearly the same {Mo_{154}} framework but with slightly different compositions in numbers of phenylalanine ligands and counter sodium cations verified by chemical analysis. It is proposed that the different compositions mainly come from the solution pH of the reaction/crystallisation conditions, thus the degree of protonation on clusters and the number of sodium cations needed for charge balances, and therefore the variable interactions between clusters that cause differences in condensation reactions and packing models. As the main clusters in all four structures are {Mo_{154}}, it is sensible to compare the volumes per {Mo_{154}} cluster in crystals which are listed in Table S1. Smaller volume per cluster indicates dense molecular packing and stronger inter-cluster interaction or more covalent bonding between clusters that means the clusters are more closely located. Note: while density is generally an important indicator of dense packing, the molecular mass required to calculate density in this context is less precise and definitive compared to the Z value. Actually the molecular mass can vary, for example when clusters are partially merged because of forming covalent bonds between clusters. Also the molecular mass is not so accurately determined, such as the TGA measurement for number of solvated water molecules. Compound **1** and **2** consist of isolated clusters connected solely by hydrogen bonding or sodium cation bridges. **1** has smaller VPC than **2**. While **1** exhibits a more efficient parallel ring arrangement of layer structure, **2** adopts less efficient packing of herringbone pattern arrangements. Compound **3** also display a herringbone pattern arrangement, but with only half {Mo_{154}} clusters remain isolated, with the other half forming 1-D chain structures through 5-folder Mo-O-Mo bridges. In contrast, Compound **4** features a parallel ring arrangement in its 2-D layer structure, with each cluster connected by four single Mo-O-Mo bridges. **4** has the smallest VPC among four structures.

Compound 1 crystallizes in monoclinic system with *C*2 space group. A half {Mo_{154}} cluster was identified in the asymmetric unit. Phenyl groups of the phenylalanine ligands were mostly well modelled and refined with partial occupancies in rigid hexagonal models. Most non-H atoms are refined anisotropically. No H atoms were added to O atoms as it is a heavy metal cluster compound. SQUEEZE procedure (from PLATON) was applied to calculate the void space and the electron counts in solvent area. Formula of compound **1** was determined by structure refinement and chemical analysis to be $Na_6H_8[Mo_{154}H_{14}O_{462}(H_2O)_{50}(C_9H_{11}NO_2)_{10}]\cdot 180H_2O$. Phenylalanine ligand is assigned to be neutral as it deprotonates at carboxylic group and protonates at amine group. Number of Mo^V centres is allocated to

be 28 with 14 fold protonation by referring to previously reported {Mo_{154}} in literature.[6] Additional 8 protons without definite location are added to the formula to balance charge. Number of solvent water molecules for the formula was assessed from structure refinement and TGA analysis. The half {Mo_{154}} cluster in the asymmetric unit extends to a complete {Mo_{154}} cluster by a crystallographic C_2 axis. **1** displays isolated clusters which have no Mo-O-Mo covalent bonding bridges between clusters. The packing diagram in Figure S4 shows layer arrangement of {Mo_{154}} wheels in solid state. Layers are displaced along the crystallographic *a* axis in repeating ABAB patterns.

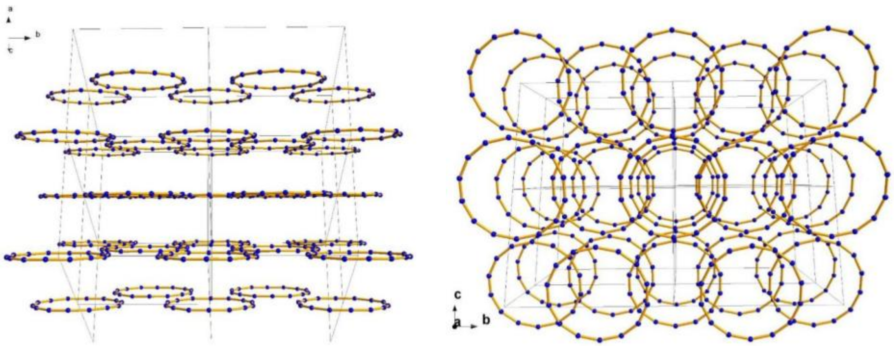

Figure S4, Representative packing diagram indicates layer arrangement of {Mo_{154}} wheels of **1** in solid state (left). View down layers of displaced ABAB patterns along the crystallographic *a* axis (right). {Mo_{154}} wheels are represented by the ring of the 14 backbone Mo centres.

Compound 2 crystallizes in orthorhombic system with $P2_12_12$ space group. Two half {Mo_{154}} clusters were found in the asymmetric unit. Most Mo sites are well defined with a few sites of in-out disorders. One {Mo_2} dimer involved Mo60 and Mo61 has only about 20% occupancy showing the defects of {Mo_{154}} cluster. Some carboxylate group of the phenylalanine ligands are roughly identified in the structure refinements. Remaining part including the phenyl group of the phenylalanine ligands were not modelled duo to too heavy disorders. Formula of compound **2** was determined to be $Na_4H_{10}[Mo_{154}H_{14}O_{462}(H_2O)_{52}(C_9H_{11}NO_2)_9]\cdot 230H_2O$ in similar manner to compound **1**. The two half {Mo_{154}} clusters in the asymmetric unit extend to two isolated {Mo_{154}} clusters each by a crystallographic C_2 axis symmetry. No connection of Mo-O-Mo bridging bonds between clusters were identified. Molecular packing shows herringbone patterns (Figure S5).

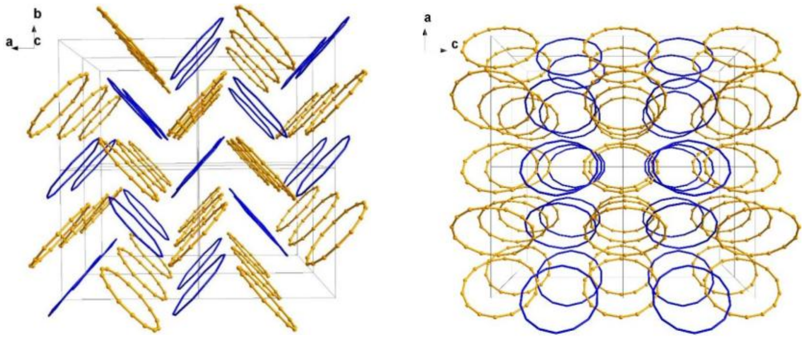

Figure S5. Representative packing diagram indicates herringbone pattern arrangement of {Mo_{154}} wheels in compound **2** viewed down the crystallographic *c* axis (left) and *b* aixs (right). Yellow and blue colours indicate {Mo_{154}} wheels from two different half {Mo_{154}} clusters found in the asymmetric unit.

Compound 3 crystallises in triclinic system with *P*-1 space group. Two half $\{Mo_{154}\}$ wheel moieties were found in the asymmetric unit. One half moiety extends to complete a normal $\{Mo_{154}\}$ wheel cluster of formula $[Mo_{154}O_{462}H_{14}(H_2O)_{56}(C_9H_{11}NO_2)_7]^{14-}$, Figure S6 above, via an inversion centre. Figure S6 below is isolated and only contacts to neighbouring $\{Mo_{154}\}$ wheels with H-bonding or Na^+ coordination in solid state. The other half moiety expands to complete an unusual $\{Mo_{154}\}$ wheel cluster with formula $[Mo_{154}O_{462}H_{14}(H_2O)_{51}(C_9H_{11}NO_2)_7]^{14-}$, Figure S6 above and below, also through an inversion centre. Figure S7 extends to form one-dimensional chain with fivefold Mo-O-Mo bridges between $\{Mo_{154}\}$ clusters. Empirical formula of compound **3** is determined as $Na_{12}H_{16}[Mo_{154}O_{462}H_{14}(H_2O)_{56}(C_9H_{11}NO_2)_7][Mo_{154}O_{462}H_{14}(H_2O)_{51}(C_9H_{11}NO_2)_7]\cdot 460H_2O$ in similar manner to compound **1**.

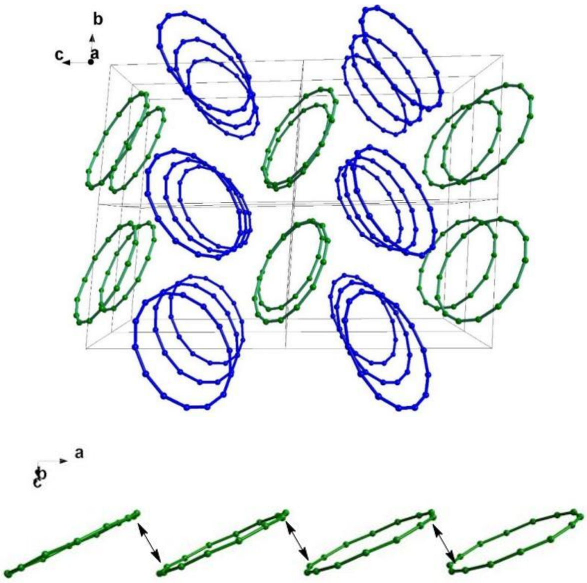

Figure S6. Above: representative packing diagram indicates columns of isolated $\{Mo_{154}\}$ wheels (blue) and 1-D chain of $\{Mo_{154}\}$ wheels (green) in compound **3**. Below: 1-D chain with dual arrows indicating the places of the fivefold Mo-O-Mo bridges between $\{Mo_{154}\}$ wheels vis orientation of the wheels.

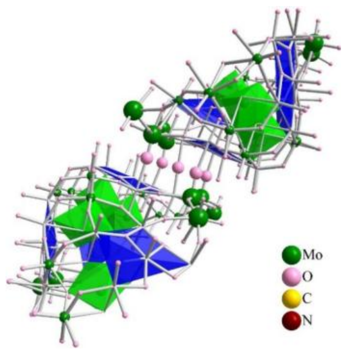

Figure S7. Local environment showing the five-fold Mo-O-Mo bridges (enlarged O spheres) between $\{Mo_{154}\}$ wheels that form 1-D chain structure.

Compound 4 crystallizes in orthorhombic system with *Aea*2 space group. One half $\{Mo_{154}\}$ cluster was found in the asymmetrical unit. Phenyl groups were partially resolved and refined. Each $\{Mo_{154}\}$ cluster links to four neighbouring $\{Mo_{154}\}$ clusters via Mo-O-Mo single bridges, therefore forming a two

dimensional layer structure of 4-connected topology (Figure S8). Formula of compound **4** was determined to be $Na_{11}H_3[Mo_{154}H_{14}O_{462}(H_2O)_{56}(C_9H_{11}NO_2)_6]\cdot 200H_2O$ in similar manner to compound **1**.

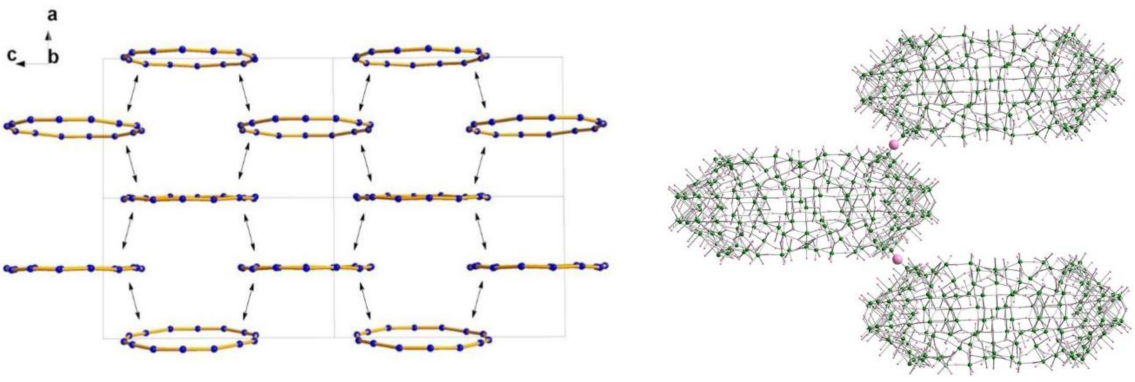

Figure S8. Representative packing diagram indicates 2-D layer structure of {Mo_{154}} wheels on the crystallographic *ac* plane in compound **4**. The dual arrow indicates the places of single Mo-O-Mo bridges between {Mo_{154}} wheels (left). Single Mo-O-Mo bridges between {Mo_{154}} wheels illustrated by enlarged O spheres (right).

7. Characterisations and Supplementary Data

Further characterisations were done on the filtered samples. Initially, the deep-blue solution was filtered along with the blue solid. The solid collected was used for elemental analysis, ICP, Uv-vis and TGA.

7.1 Chemical analysis

Table S11. Results of elemental analysis for C, H, N and ICP for Mo, Na

	C%		H%		N%		Mo%		Na%	
	Calc.	Found	Calc.	Found	Calc.	Found	Calc.	Found	Calc.	Found
**1**	3.84	3.62	2.12	1.83	0.50	0.61	52.54	53.28	0.49	0.49
**2**	3.37	3.01	2.40	2.66	0.44	0.47	51.22	52.38	0.32	0.31
**3**	2.65	2.27	2.35	2.13	0.34	0.27	51.68	52.6	0.48	0.43
**4**	2.31	2.35	2.14	1.78	0.30	0.39	52.70	53.9	0.90	0.92

7.2 Uv-vis Spectra

All the Uv-vis spectra of **1-4** show the characteristic band of Mo Blue which is centered around 751 nm.

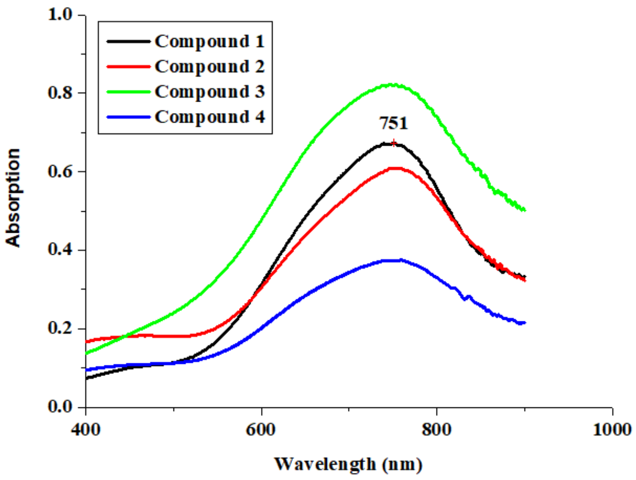

Figure S9. Uv-vis spectra of compound 1-4.

7.3 Thermogravimetric analysis

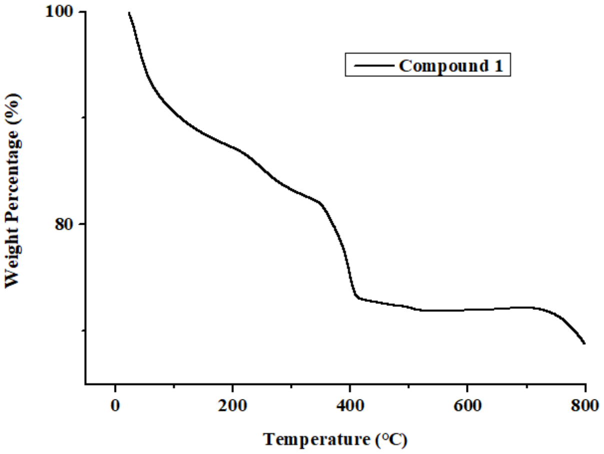

Figure S10. TGA curve of compound **1**. 11.5% weight loss between r.t. and 200 °C corresponds to ~180 crystalline H_2O, further 13.6% weight loss from 200 to 400 °C corresponds to 10 L-phenylalanine ligands and water ligands on cluster.

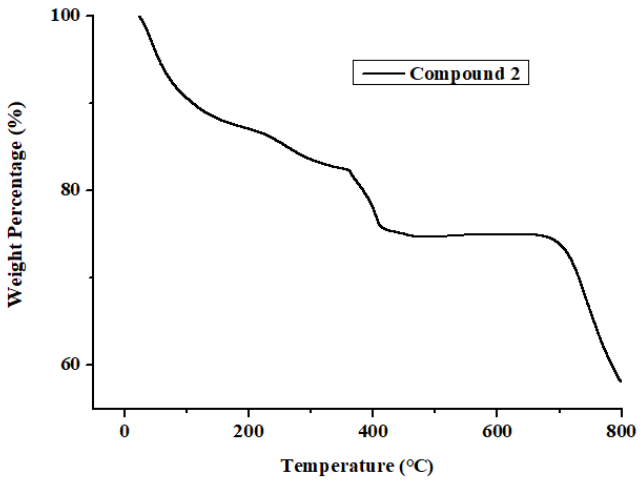

Figure S11. TGA curve of compound **2**. 14.3% weight loss between r.t. and 200 °C corresponds to ~230 crystalline H_2O, further 10.2% weight loss from 200 to 400 °C corresponds to 9 L-phenylalanine ligands and water ligands on cluster.

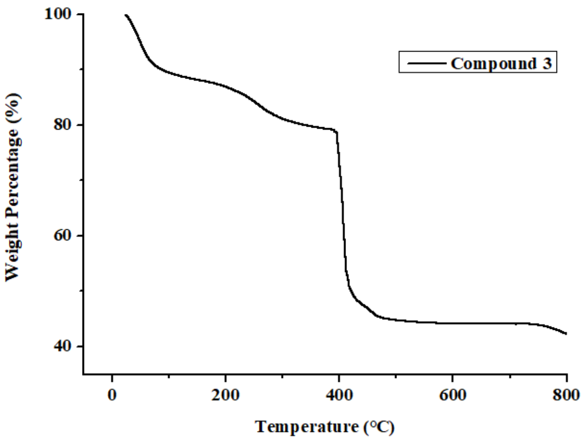

Figure S12. TGA curve of compound **3**. 14.5% weight loss between r.t. and 150 °C corresponds to ~460 crystalline H_2O. Nearly 13.2% weight loss weight loss at 400 °C. It may mean there was an explosion at 400 °C and some Mo oxide also lost.

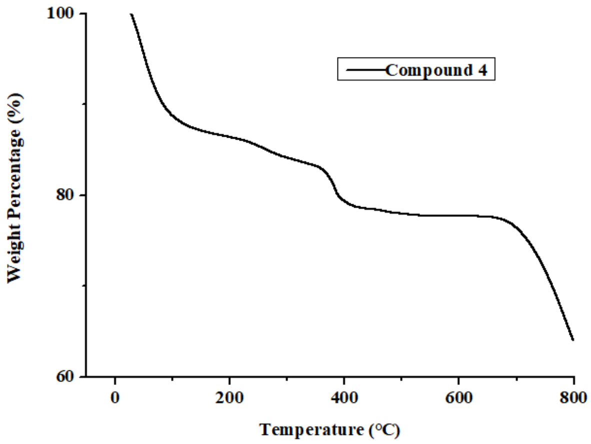

Figure S13. TGA curve of compound **4**. 12.9% weight loss between r.t. to 200 °C corresponds to ~200 crystalline H_2O, further 7.8% weight 200 to 400 °C corresponds to 6 L-phenylalanine ligands and water ligands on cluster.

7.4 FT- IR Spectroscopy

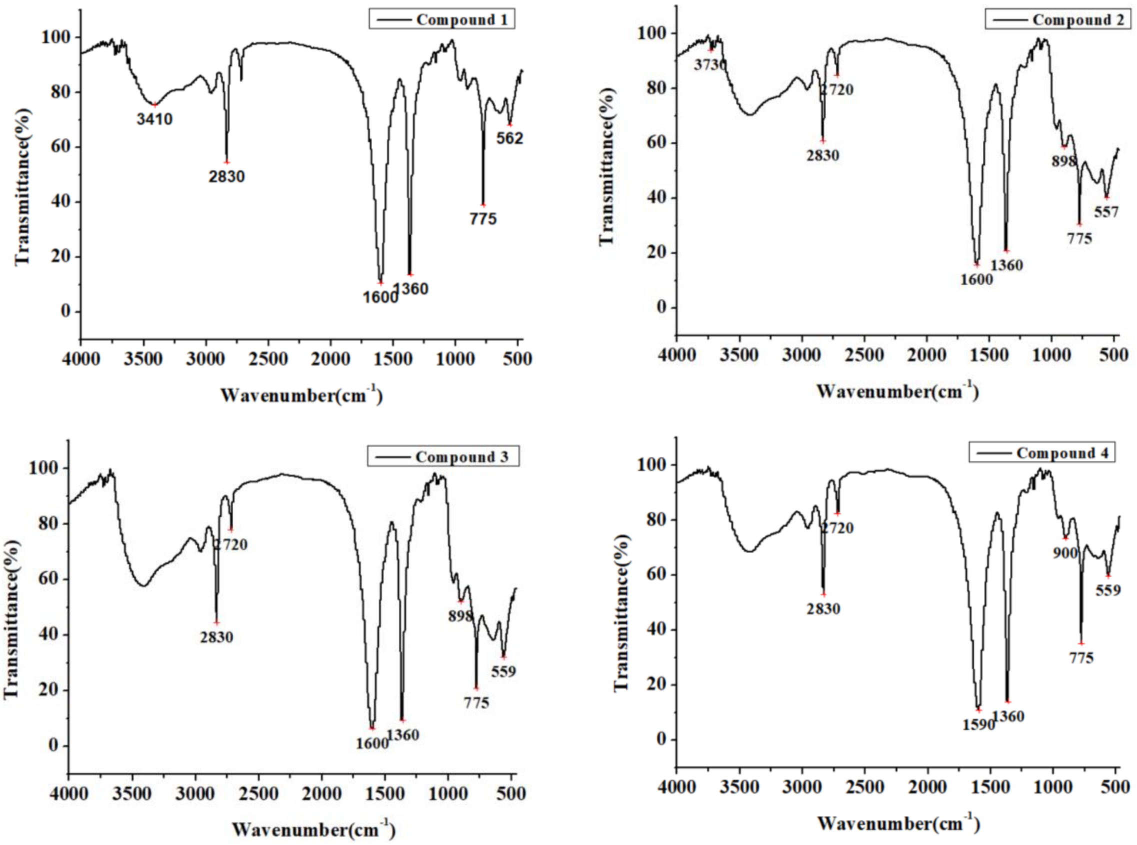

Figure S14. IR spectra of compound **1-4**.

8. References

1 G. M. Sheldrick, *Acta Crystallogr. Sect. A: Found. Crystallogr.*, 2015, **71**, 3-8.
2 G. M. Sheldrick, *Acta Crystallogr. Sect. C: Cryst. Struct. Commun.*, 2015, **71**, 3-8.
3 L. J. Farrugia, *J. Appl. Crystallogr.*, 1999, **32**, 837-838.
4 A. L. Spek, *J. Appl. Crystallogr.*, 2002, **36**, 7-13.
5 W. R. Busing and H. A. Levy, *Acta Crystallographica*, 1957, **10**, 180-182.
6 A. Muller, J. Meyer, E. Krickemeyer and E. Diemann, *Angew. Chem. Int. Ed. Engl.*, 1996, **35**, 1206-1208.
